# Integration of Dual Stress Transcriptomes and Major QTLs from a Pair of Genotypes Contrasting for Drought and Chronic Nitrogen Starvation Identifies Key Stress Responsive Genes in Rice

**DOI:** 10.1186/s12284-021-00487-8

**Published:** 2021-06-05

**Authors:** Amitha Mithra Sevanthi, Subodh Kumar Sinha, Sureshkumar V, Manju Rani, Manish Ranjan Saini, Sapna Kumari, Megha Kaushik, Chandra Prakash, Venkatesh K., G. P. Singh, Trilochan Mohapatra, Pranab Kumar Mandal

**Affiliations:** 1 ICAR-National Institute for Plant Biotechnology, Pusa Campus, New Delhi, 110012 India; 2 ICAR-Indian Institute of Wheat and Barley Research, Karnal, 132001 India; 3 Indian Council of Agricultural Research, Krishi Bhavan, New Delhi, 110001 India

**Keywords:** Nitrogen stress, Drought stress, Rice, QTLs under nitrogen stress, RNA-seq, Nitrogen use efficiency

## Abstract

We report here the genome-wide changes resulting from low N (N-W+), low water (N+W-)) and dual stresses (N-W-) in root and shoot tissues of two rice genotypes, namely, IR 64 (IR64) and Nagina 22 (N22), and their association with the QTLs for nitrogen use efficiency. For all the root parameters, except for root length under N-W+, N22 performed better than IR64. Chlorophyll a, b and carotenoid content were higher in IR64 under N+W+ treatment and N-W+ and N+W- stresses; however, under dual stress, N22 had higher chlorophyll b content. While nitrite reductase, glutamate synthase (GS) and citrate synthase assays showed better specific activity in IR64, glutamate dehydrogenase showed better specific activity in N22 under dual stress (N-W-); the other N and C assimilating enzymes showed similar but low specific activities in both the genotypes. A total of 8926 differentially expressed genes (DEGs) were identified compared to optimal (N+W+) condition from across all treatments. While 1174, 698 and 903 DEGs in IR64 roots and 1197, 187 and 781 in N22 roots were identified, nearly double the number of DEGs were found in the shoot tissues; 3357, 1006 and 4005 in IR64 and 4004, 990 and 2143 in N22, under N-W+, N+W- and N-W- treatments, respectively. IR64 and N22 showed differential expression in 15 and 11 N-transporter genes respectively, under one or more stress treatments, out of which four showed differential expression also in N+W- condition. The negative regulators of N- stress, e.g., *NIGT1*, *OsACTPK1* and *OsBT* were downregulated in IR64 while in N22, *OsBT* was not downregulated. Overall, N22 performed better under dual stress conditions owing to its better root architecture, chlorophyll and porphyrin synthesis and oxidative stress management. We identified 12 QTLs for seed and straw N content using 253 recombinant inbred lines derived from IR64 and N22 and a 5K SNP array. The QTL hotspot region on chromosome 6 comprised of 61 genes, of which, five were DEGs encoding for UDP-glucuronosyltransferase, serine threonine kinase, anthocyanidin 3-O-glucosyltransferase, and nitrate induced proteins. The DEGs, QTLs and candidate genes reported in this study can serve as a major resource for both rice improvement and functional biology.

**Supplementary Information:**

The online version contains supplementary material available at 10.1186/s12284-021-00487-8.

## Background

Nitrogen (N) is one of the most important macronutrients of plants as it is a component of several biomolecules, viz., enzymes, amino acids, nucleic acids, chlorophyll, and a range of diverse secondary metabolites. Thus, it affects many aspects of plant growth and metabolism and eventually the grain yield (Xu et al. 2012; Wang et al. 2014; Sinha et al. 2019). Enhanced N supply combined with enhanced N responsiveness of the semi-dwarf genotypes of the major cereal crops, especially, rice and wheat, made higher harvest index and productivity possible which eventually ushered in the green revolution (Zeigler and Mohanty, 2010). Though both semi-dwarf rice and wheat respond to N fertilizers, they have low N use efficiency and thereby demand higher supply of N for higher yields (Loddo and Gooding, 2012; Ma and Liu, 2018). It has been estimated that crop plants in general are capable of utilizing only 30–40% of applied N (Raun and Johnson, 1999), and hence more than 60% applied N is lost to the environment. However, continuous and/ or indiscriminate supply of nitrogenous fertilizers had not only led to economic losses to farmers but also has caused severe environmental pollution as a result of the numerous physio-chemical processes that occurs in the soil, e.g., eutrophication of freshwater and the marine ecosystem due to leaching and run-off of excessive NO_3_^−^, volatilization and denitrification (Snyder et al. 2009). Further, the emission of N_2_O (a greenhouse gas which is almost 300 times more potent than CO_2_) has become a major threat to our environment (Forster et al., 2007).

Global warming is posing a grave threat to water supply with irregular quantum and periodicity of rainfall that has made intermittent drought spells during rice cultivation a common occurrence. Under the rainfed system of rice cultivation, drought stress is a major cause of yield loss (Prasertsak and Fukai, 1997; Dar et al. 2020). Though, the irrigated system is the major form of rice cultivation in India and China, the two major rice growing countries of the world, this will arguably become unsustainable by the second half of twenty-first century, owing to global warming led depletion of water resources. Further, the semi-dwarf genotypes of rice, that occupy the major area of rice fields, are highly sensitive to drought stress as there is a tight linkage between the *sd1* gene and a major drought tolerant quantitative trait loci (QTL) on chromosome 1 (Vikram et al. 2015). Thus, poor supply of N and water are the major limiting factors for plant growth and crop productivity (Ding et al. 2018).

Root, being the first organ of a plant, senses the availability of N and moisture in the soil besides other minerals and often gets modulated in resource-limited conditions, e.g., low N and water, probably to acquire these nutrients (Sinha et al. 2020). Drought stress negatively impacts plant growth and a plethora of other physiological processes among which the most important ones are C (carbon) and N metabolism (Abid et al. 2016). Owing to cell membrane damage under drought stress, N transport and its metabolism are reduced in plants (Xu et al. 2006; Xiong et al. 2018). Rice being traditionally grown in flooded condition, prefers ammonical-N rather than nitrate N, and the former was demonstrated to enhance drought tolerance in rice through the expression of aquaporins and accumulation of ABA (Guo et al. 2007; Gao et al. 2010; Ding et al., 2016 and 2018). However, due to the presence of aerenchyma cells in the root, rice is capable of transporting oxygen from the leaf to the rhizosphere enabling oxidation of NH_4_^+^ to NO_3_^−^, and therefore can take up 15–40% of N in the form of NO_3_^−^ (Kirk and Kronzucker, 2005). Nitrate transporters belonging to NPF family (consisting members of low affinity nitrate transporters) have been demonstrated to transport a variety of substrates including ABA and IAA (Leran et al. 2014). Further, overexpression of an accessory protein of a high affinity nitrate transporter of rice, *OsNAR2.1*, has been demonstrated to not only increase N use efficiency and yield but also drought tolerance while the RNAi lines had lower drought tolerance (Chen et al. 2016, 2019). They also showed that the *OsNRT2.1* and *OsNRT2.3a* transgenic lines did not provide any advantage under drought stress and rather had a very low survival rate. ABA is the key player in several abiotic stress tolerance mechanisms including drought and salinity tolerance (Peleg and Blumwald, 2011). Osmotic stress/ABA–activated protein kinase 2 (*SAPK2*), a member of SnRK2s subclass II, has been shown to enhance NUE under drought stress (Lou et al. 2020). Higher N supply is also known to predispose plants to pest and disease attack, and downregulate ABA levels (Cao et al. 2011). Thus, crosstalk among N transporters, ABA dependent gene expression and aquaporins including nodulin 26-like intrinsic protein family (which are a super class of plant aquaglyceroporins), under adequate or higher N supply, may reduce the negative impact of drought stress in plants or vice-versa (Ding et al. 2018). At the field level also, N fertilizer application had a small effect on growth during the drought stress period in rice, while a larger positive effect of N was observed after recovery in the stress trials. On the other hand, there are some metabolites, for instance, allantoin, which show exactly opposite fates under limited N and water supply stress. Allantoin showed higher catabolism under N-limited conditions, and on the contrary, accumulation of allantoin was seen under the water stress conditions in two Australian bread wheat genotypes (Casartelli et al., 2019). Further, allantoin is reported to be accumulated under drought stress in drought sensitive genotypes in legumes but drought tolerant genotypes in cereal crops (Plett et al. 2020).

With the advent of microarray and RNA-seq technologies, individual effects of suboptimal supply of N and water on the entire physiology and gene expression have been examined in detail by many workers (Lian et al. 2006; Cai et al. 2012; Yang et al. 2015; Sinha et al. 2018; Li et al. 2019; Song et al. 2020). The interplay of N and water use efficiency is an important aspect of crop production, and therefore their dissection at the molecular level is highly imperative. To the best of our knowledge, genome-wide transcriptome studies on their combined deficiency effect have not been reported so far in rice or other crop plants. Hence, in order to understand the effect of dual stress (low N and water supply stress), we examined a global transcriptome of root and shoot tissues of rice seedlings of two rice genotypes, i.e., Nagina 22 (a drought tolerant, tall and non-N responsive upland variety), and IR 64 (a semi-dwarf, drought sensitive and N-responsive lowland variety), employing physio-biochemical characterization of these two contrasting genotypes for individual and dual stresses. We further identified low-N stress specific QTLs using a recombinant inbred (RI) population derived from these two parents. Our results suggest that IR64 had a similar molecular response to N- and dual (N-W-) stress, while the response of N22 under these two conditions was distinctly different. Though both the genotypes did show downregulation of the major metabolic pathways, the degree of downregulation was consistently lower in N22 as compared to IR64 under dual stress. We also could identify some major putative candidate genes for higher nitrogen use efficiency through integration of molecular mapping and transcriptome data under N-stress.

## Results

### Effect of Low Nitrogen and Water Stress on Biomass

**Fig. 1:**
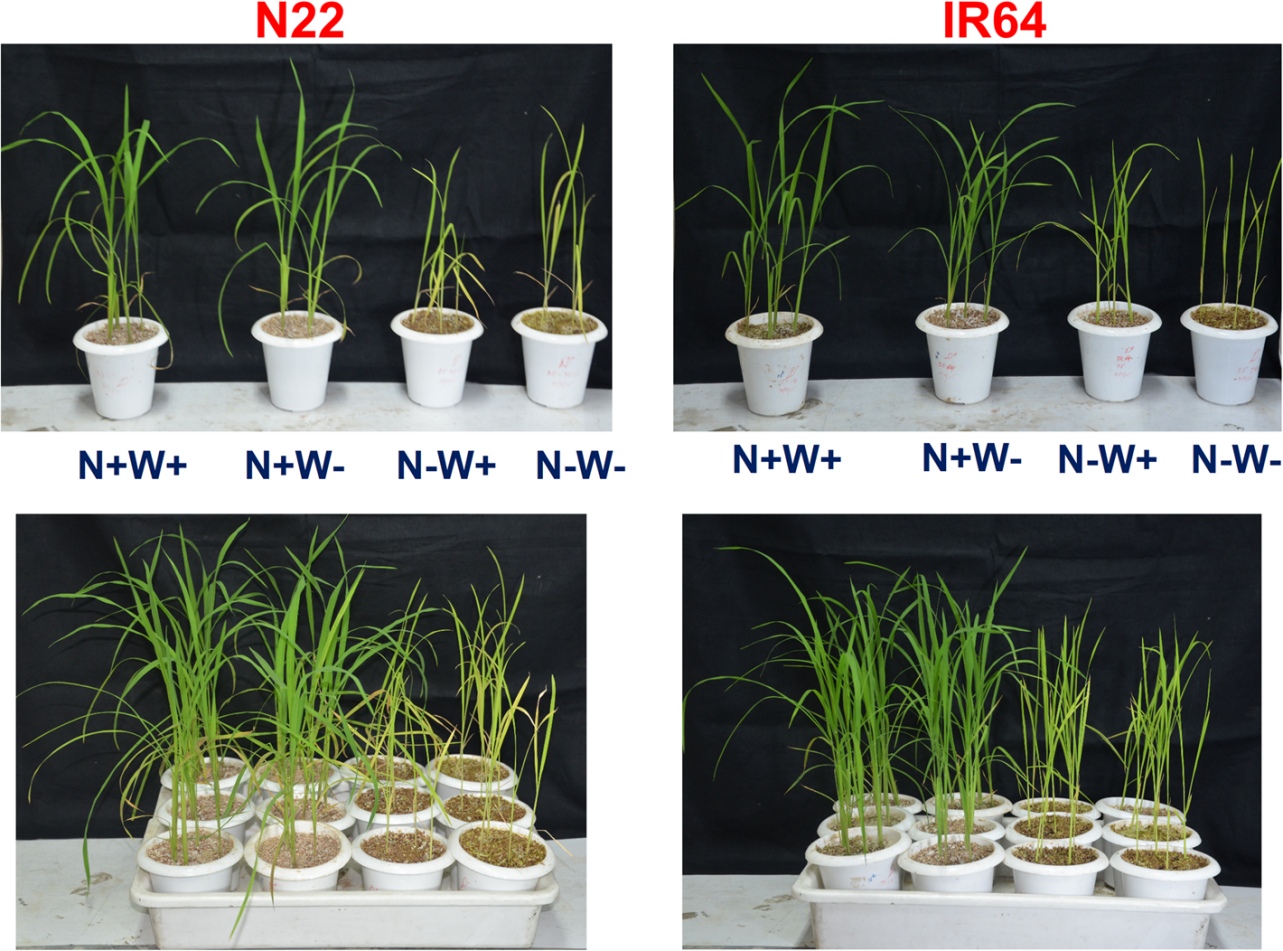
Image of the plants grown under various stress treatments namely optimal input (N+W+), low N (N-W+), low water (N+W-) and low N and water (N-W-) supply

**Fig. 2:**
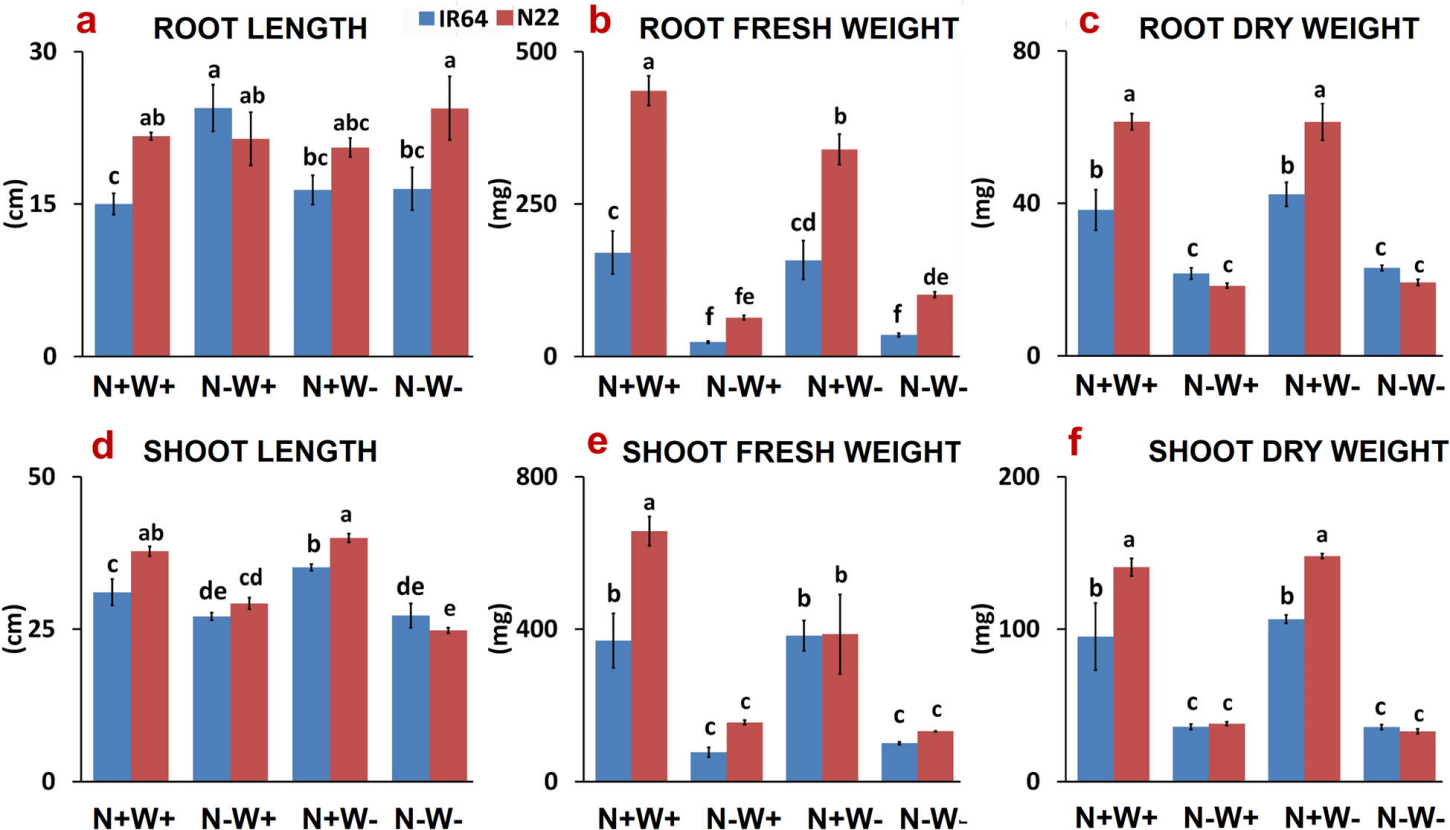
Biomass (Root and Shoot Length; Root and Shoot Fresh Weight and Root and Shoot Dry Weight) analysis of two rice genotypes, i.e., IR64 and N22, under low nitrogen (N) and water (W) stresses. Growth conditions followed by plus (+) and minus (−) indicate optimal (+) and low (−) supply of input. Values are mean ± SE (n = 3), different letters above the bar indicate significant difference (*p* < 0.05) between the different stress conditions and genotypes

The plants subjected to all the three stress treatments along with the optimal conditions are shown in Fig. 1. The longest root length was observed in IR64 (24.46 cm) under limited N supply (N-W+) followed by N22 (24.43 cm) under dual stress (N-W-) treatment. The shortest root length of 15 cm was observed under N+W+ in IR64. In general, the root length of N22 was significantly longer as compared to IR64 in all conditions except N-W+ (Fig. 2a). Root fresh weight (mg) of N22 was much higher than that of IR64 under optimal as well as all the stress conditions, i.e., 436.26 vs. 170.56 (N+W+), 63.8 vs. 23.6 (N-W+), 339.83 vs. 158.16 (N+W-) and 101.5 vs. 35.2 (N-W-) (Fig. 2b). While both the genotypes showed similar reduction for root dry weight under N-W+ stress and N-W- conditions, N22 had higher root dry weight under optimal and N+ W- stress condition than that of IR64 (Fig. 2c). Under N-W+ condition, IR64 showed longer root length and maintained both its root dry and fresh weight, whereas N22 maintained its root length but showed severe reduction in root weight (Fig. 2a-c). Thus, under N-W+ and dual stress (N-W-), though the two genotypes showed a similar performance for root fresh and dry weight, in terms of % reduction in root weight, N22 showed a higher reduction compared to IR64 (43.47% vs. 70.02% reduction under N-W+ stress and 38.63 vs. 68.62% reduction under N+W- in root dry weight in IR64 and N22 respectively); however, under N+W- stress, the performance of the genotypes was similar as evident from a similar % reduction in root and shoot dry weights (− 5.7 vs. 0.16% for RDW and − 12.05 vs. -5.17%).

Shoot length of N22 was the longest under N+W- condition (39.9 cm), equivalent to optimal conditions, and invariably higher than that of IR64 under optimal as well as all stress conditions (Fig. 2d). The only exception to this was the dual stress condition wherein both the genotypes had nearly equal shoot length. Further, under optimum conditions (N+W+), both shoot fresh and dry weight of N22 were significantly higher than that of IR64, i.e., 657.06 mg and 140.5 mg as compared to 369.9 mg and 95.13 mg respectively. Both the genotypes showed significantly reduced shoot weight under stress conditions and were equivalent under all the cases except N+W- condition wherein N22 had more shoot dry weight than IR64 (Figs. 2e-f). In brief, the resilience IR64 to chronic N-W+ stress was evident from the results while N22 and IR64 showed a nearly similar performance under a short term N+W- stress.

### Effect of Low Nitrogen and Water Stress on Relative Water Content (RWC)

**Fig. 3:**
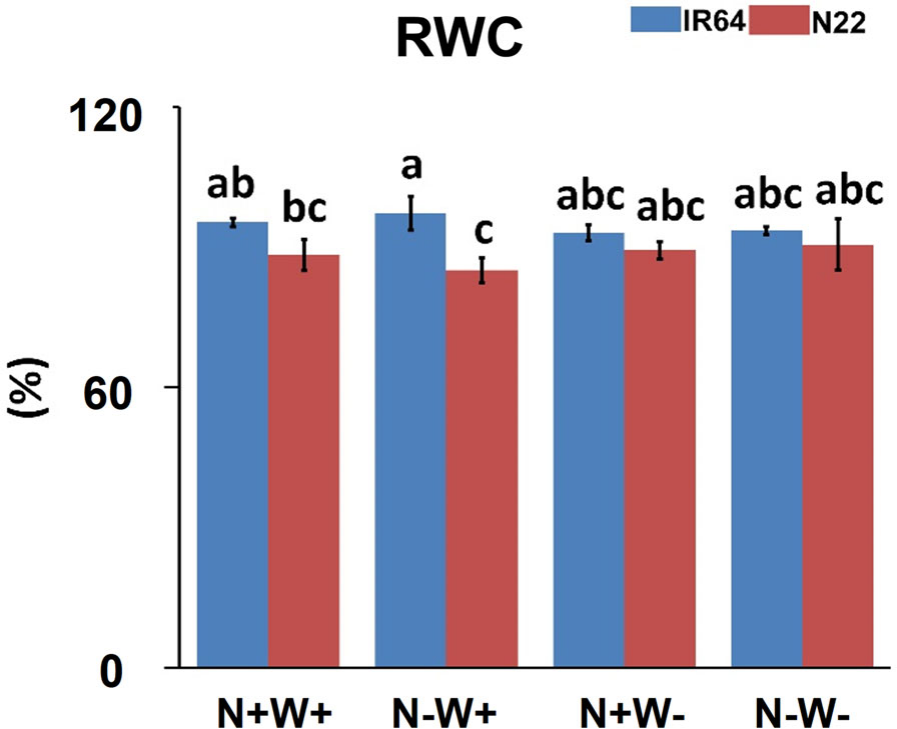
Relative Water Content (RWC) of two rice genotypes, i.e., IR64 and N22, under low nitrogen (N) and water (W) stresses. Growth conditions followed by plus (+) and minus (−) indicate optimal (+) and low (−) supply of input. Values are mean ± SE (*n* = 3), different letters above the bar indicate significant difference (*p* < 0.05) between the different stress conditions and genotypes

Both the highest and lowest RWC (%) was observed under N-W+ condition in IR64 (97.2%) and N22 (84.24%) respectively. Differences between the genotypes for RWC was not significant under the dual stress (N-W-), i.e., 93.39 and 90.4% for IR64 and N22 respectively. However, IR64 showed better RWC than that of N22 under N-W+ stress condition with intermediate values in other treatments (Fig. 3). Notably, under the dual stress, the RWC was even more than that of N+W- stress in N22 while it was nearly equivalent in IR64. It’s important to mention here that these differences were however not statistically significant.

### Effect of Low Nitrogen and Water Stress on Chlorophyll and Carotenoid Content

**Fig. 4:**
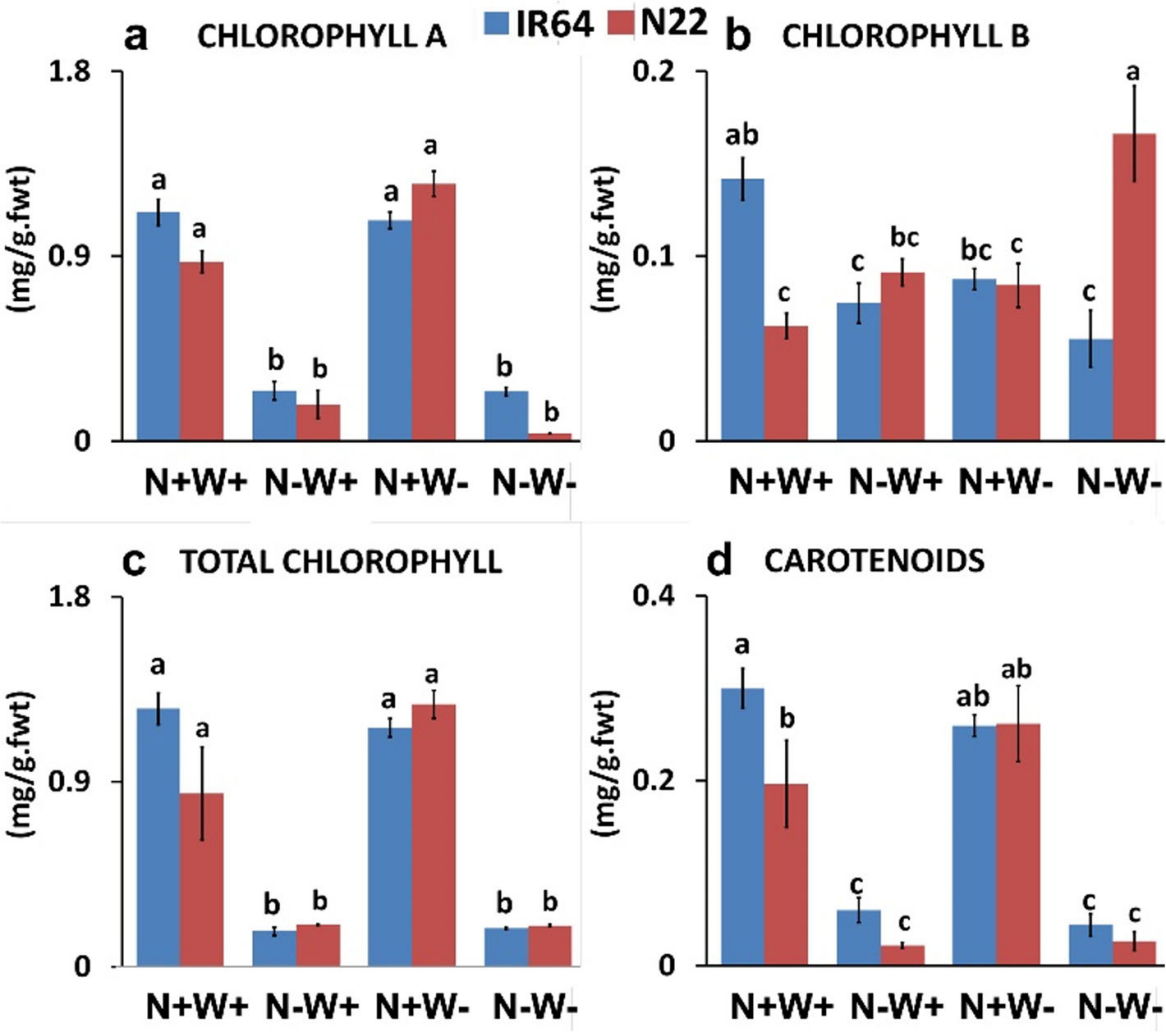
Chlorophyll and carotenoid content of two rice genotypes, i.e., IR64 and N22, under low nitrogen (N) and water (W) stresses. Growth conditions followed by plus (+) and minus (−) indicate optimal (+) and low (−) supply of input. Values are mean ± SE (n = 3), different letters above the bar indicate significant difference (*p* < 0.05) between the different stress conditions and genotypes

Reduction in chlorophyll A, total chlorophyll and carotenoid content was observed only under N-W+ stress or dual stress (N-W-) but not under W- stress in both the genotypes. So it was only the Chl B content that distinguished the response of the genotypes under optimum as well as dual stress conditions. N22 showed both maximum (0.166 mg/ g.fwt) and minimum (0.0622 mg/ g.fwt) content of Chl B under N-W-, and N+W+ conditions, respectively, whereas IR64 had the highest Chl B content, i.e., 0.1418 mg/ g.fwt under optimum conditions. Although stress conditions did cause reduction of Chl A and total Chl, the difference between the genotypes was insignificant under both optimum as well as stress conditions (Figs. 4a-c). Carotenoid content was higher in IR64 under the optimal condition. Though RWC content did not show much variation under W- and dual stress in either of the genotypes (Fig. 3), the leaves showed complete rolling and yellowing, especially, under dual stress, as seen from the very low chlorophyll content, especially Chl A in N22 and Chl B in IR64 (Figs. 1 and 4). Hence, sampling was done on the 7th day of drought stress for further morphological, biochemical and transcriptome studies.

### Effect of Low Nitrogen and Water Stress on Root System Architecture Traits

**Fig. 5:**
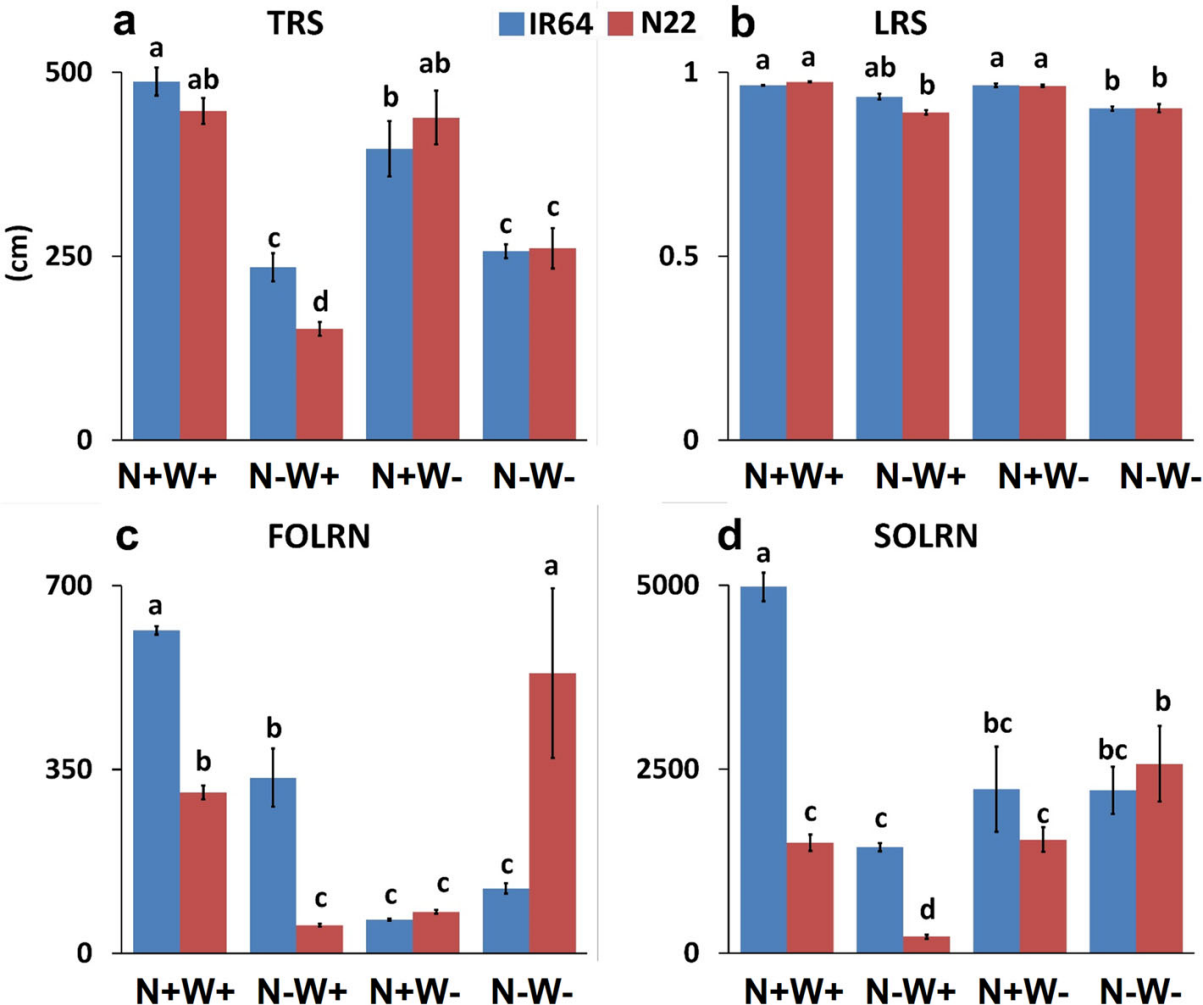
Root system architecture (RSA) of two rice genotypes, i.e., IR64 and N22, under low nitrogen (N) and water (W) and combined stresses. Growth conditions followed by plus (+) and minus (−) indicate optimal (+) and low (−) supply of input. Values are mean ± SE (n = 3), different letters above the bar indicate significant difference (*p* < 0.05) between the different stress conditions and genotypes. TRS: Total root size; LRS: lateral root size; FOLRN: first order lateral root number; SOLRN: Second order lateral root number

**Fig. 6:**
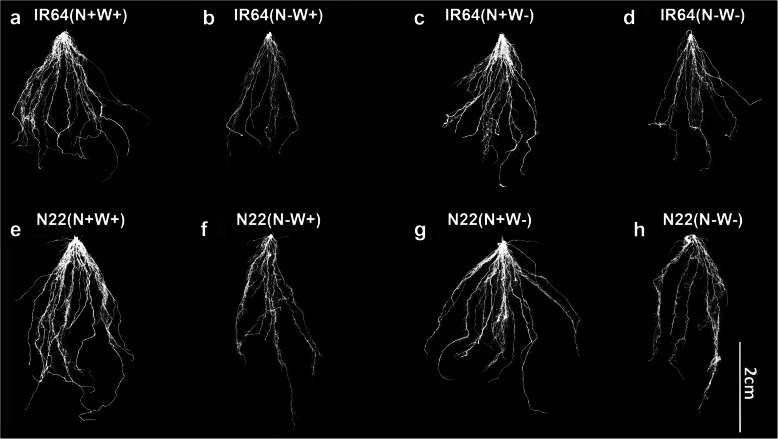
Root system architecture images of the two genotypes, IR64 and N22, under low nitrogen (N) and water (W) and combined stresses. Growth conditions followed by plus (+) and minus (−) indicate optimal (+) and low (−) supply of input

Under optimal (N+W+) and N-W+ conditions, the TRS of IR64 (487.55 and 235.03 cm respectively) was observed to be more than N22 (447.88 and 151.64 cm respectively; Figs. 5a and Fig. 6). In brief, the genotypes did not show a statistically significant difference between them for TRS under optimal input supply, but did show a significantly differential response under low N- supply i., N-W+ stress. Similarly, TRS got reduced significantly in IR64 under N+W- stress (487.55 to 396.46 cm), while only a marginal reduction (447.88 to 438.91 cm) was seen in N22 (Figs. 5a and Fig. 6). Under dual stress conditions (N-W-), the TRS of both the genotypes reduced considerably but the difference between the genotypes was again statistically not significant. LRS was not affected by N+W- stress in both the genotypes. The least LRS was observed under dual stress condition in both the genotypes (0.9023 and 0.9036 for IR64 and N22 respectively), followed by N-W+ condition, wherein only N22 showed reduction (0.8920) but not IR64 (0.9340) (Fig. 5b). In general, SOLRN was more than FOLRN in both the genotypes under respective conditions. IR64 showed highest FOLRN (614.66) and SOLRN (4979.33) under the optimal condition, but showed a comparatively severe reduction under all the stress conditions (Figs. 5c-d). N22 showed an interesting pattern of lateral root numbers, wherein FOLRN showed the maximum value under dual stress condition (533.3), followed by optimal (306.0), W- (79.0) and N- (54.0) conditions. Similarly in case of SOLRN, N22 showed the highest value (2574.0) under dual stress conditions, followed by optimal (1499.0), W- (1541.0) and N- (222.33) conditions, the latter being the least number among all conditions and genotypes (Figs. 5d and Fig. 6).

### Effect of Low Nitrogen and Drought Stress on N and C Metabolizing Enzymes

**Fig. 7:**
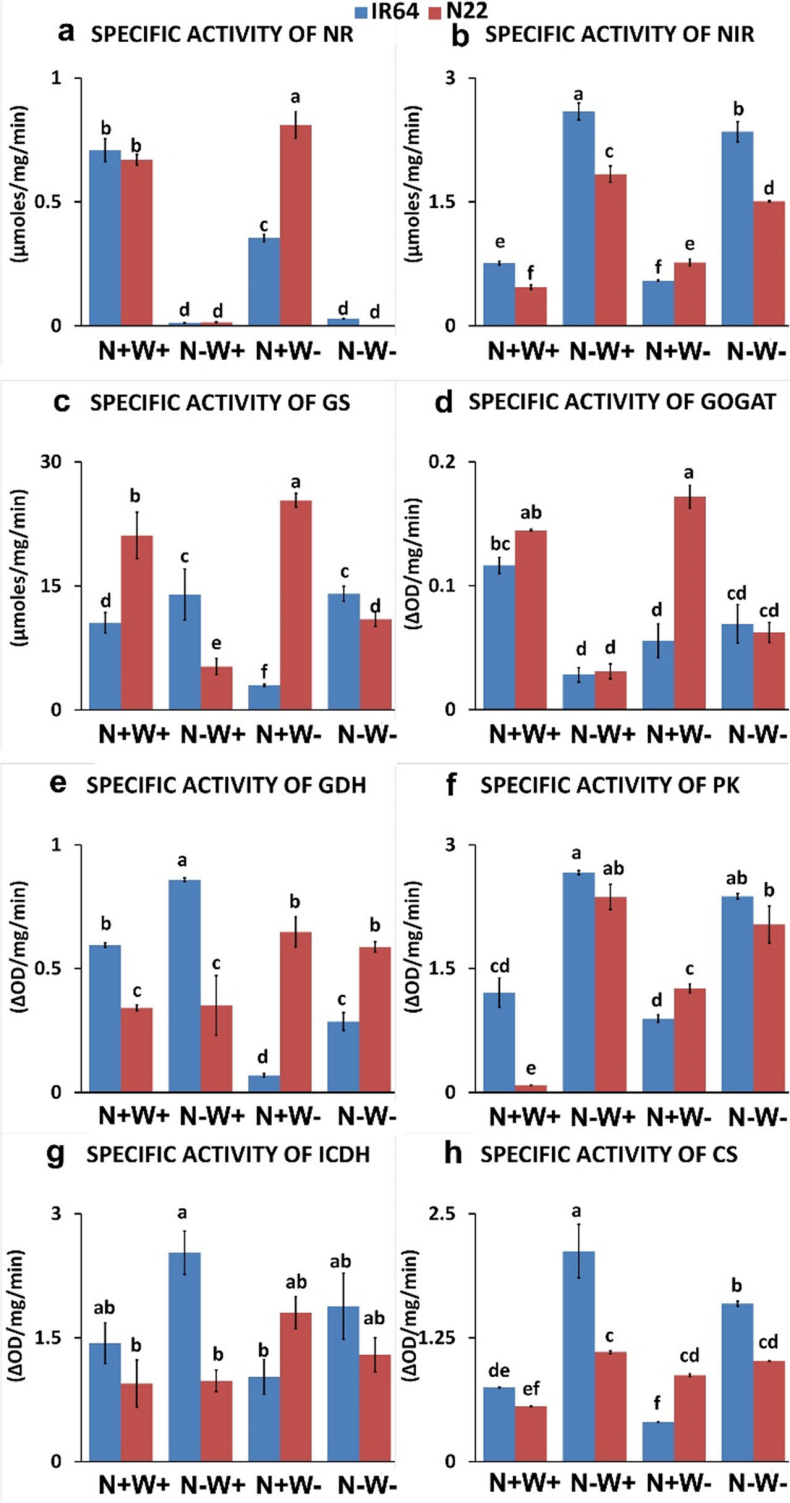
Specific activities of N and C-metabolizing enzymes (NR, NiR, GS, GOGAT, GDH, PK, ICDH and CS) of two rice genotypes, i.e. IR64 and N22, under nitrogen (N) and water (W) stress. Growth conditions followed by plus (+) and minus (−) indicate optimal (+) and low (−) supply of input. Values are mean ± SE (n = 3), different letters above the bar indicate significant difference (*p* < 0.05) between the different stress conditions and genotypes. NR: Nitrate reductase; NiR: Nitrite reductase; GS: Glutamine synthase; GOGAT: glutamate-oxoglutarate aminotransferase; GDH: Glutamate dehydrogenase; PK: Pyruvate kinase; ICDH: Isocitrate dehydrogenase; CS: Citrate synthase

Maximum specific activity (μmoles/mg/min) of NR was found in N22 under N+W- condition, i.e., 0.81, followed by N+W+ condition in both the genotypes (0.70 in IR64 and 0.67 in N22) and N+W- conditions in IR64 (0.35). The least NR activity was observed in both N-W+ and N-W- conditions for both genotypes (Fig. 7). Interestingly, NiR activity was always higher under N- stress i.e., both N-W+ (2.59 and 1.83 μmoles/mg/min for IR64 and N22 respectively) and N-W- conditions (2.35 and 1.50 μmoles/mg/min for IR64 and N22 respectively). N22 showed the highest activities of GS and GOGAT in N+W- condition, i.e., 25.33 μmoles/mg/min and 0.17 ∆OD/mg/min, as compared to other conditions and IR64 genotype. On the contrary, IR64 showed highest activity of GDH under N-W+ condition (0.85 ∆OD/mg/min) and least activity in N+W- condition (0.06 ∆OD/mg/min) as compared to the other conditions and N22 genotype. Following a similar trend, IR64 showed highest activity for all the three C-metabolizing enzymes, i.e., PK (2.66 ∆OD/mg/min), ICDH (2.52 ∆OD/mg/min) and CS (2.12 ∆OD/mg/min) under N-W+ condition. As far as these three C-metabolizing enzymes are concerned, IR64 showed minimum activities in N+W- condition, whereas N22 showed minimum activity under N+W+ condition (Fig. 7). Overall, under dual stress (N-W-), NiR, PK and CS enzyme assays showed significantly better specific activity in IR64, while most of the other N and C assimilating enzymes showed similar but low specific activities in both the genotypes. When the enzyme activities under dual stress were compared with that of optimal input supply, four of the eight enzymes, NiR, PK, ICDH and CS, showed better specific activity under dual stress whereas NR and GOGAT showed lower specific activity. In case of GS and GDH, IR64 had better specific activity under dual stress for the former and N22 had better specific activity for the latter in comparison to optimal input supply. Under drought stress, N22 performed better than IR64 for all the eight enzymes assayed, while under the optimal conditions IR64 performed better than N22 except for GS.

### Genome-Wide Transcriptome Sequencing under Low Nitrogen and Water Stress – Data Quality and Identification of DEGs

A total of 1.16 billion raw reads were obtained across 16 treatments with a range of 43.95 (IR 64 shoot under N-W+) to 85.54 M reads (N22 root under N+W+), and an average of 72.58 M raw reads per treatment (Table 1). On an average, 66.51 M (91.37%) high quality reads were available for further analyses after trimming of low quality reads. Though the N specific transcriptomes (N+W+ and N-W+) were previously reported by us (Sinha et al. 2018), we had used open source algorithms and could map only 47.35% of the reads to the reference genome. However, in the present study, 89.91% of the reads could be mapped to the genome as we used comparatively relaxed parameters available in the CLC workbench (Table 1). This was essential because N22, an *aus* type, and IR64, an *indica* type of rice, are quite distinct from the reference genome of Nipponbare (*japonica* type).

**Table 1:** Summary of transcriptome data obtained for low nitrogen (N) and water stress response studies in a pair of rice genotypes, IR 64 and Nagina 22 (N22)

Treatment	Library	Number of raw reads	Number of high quality (HQ)	% of HQ reads	Mapping % of HQ reads
A: Optimal N and water (N + W+)	IR64R	66,536,324	50,191,956	75.45	88.81
	IR64S	70,824,528	55,136,806	77.85	91.98
B: Low N and optimal water (N-W+)	IR64R	82,505,318	79,630,496	96.52	84.57
	IR64S	80,815,020	75,536,524	93.47	92.84
C: Optimal N and low water (N + W-)	IR64R	71,789,678	65,991,386	91.92	93.66
	IR64S	59,320,262	54,086,464	91.18	92.58
D: Low N and low water (N-W-)	IR64R	83,704,236	78,718,332	94.04	92.58
	IR64S	43,945,150	38,574,194	87.78	92.8
A: Optimal N and water (N + W+)	N22R	85,535,858	78,152,624	91.37	93.98
	N22S	80,808,216	74,622,520	92.35	83.15
B: Low N and optimal water (N-W+)	N22R	80,715,748	76,311,074	94.54	94.04
	N22S	64,090,592	62,037,382	96.80	84.51
C: Optimal N and low water (N + W-)	N22R	68,228,978	64,247,540	94.16	92.72
	N22S	72,947,448	67,688,471	92.79	80.84
D: Low N and low water (N-W-)	N22R	76,438,072	72,888,018	95.36	91.01
	N22S	73,075,028	70,427,288	96.38	88.51
Total reads		1,161,280,456	1,064,241,075	91.37	89.91
Average reads per library		72,580,028.5	66,515,067.19		

Note: *R* root; *S* shoot

**Fig. 8:**
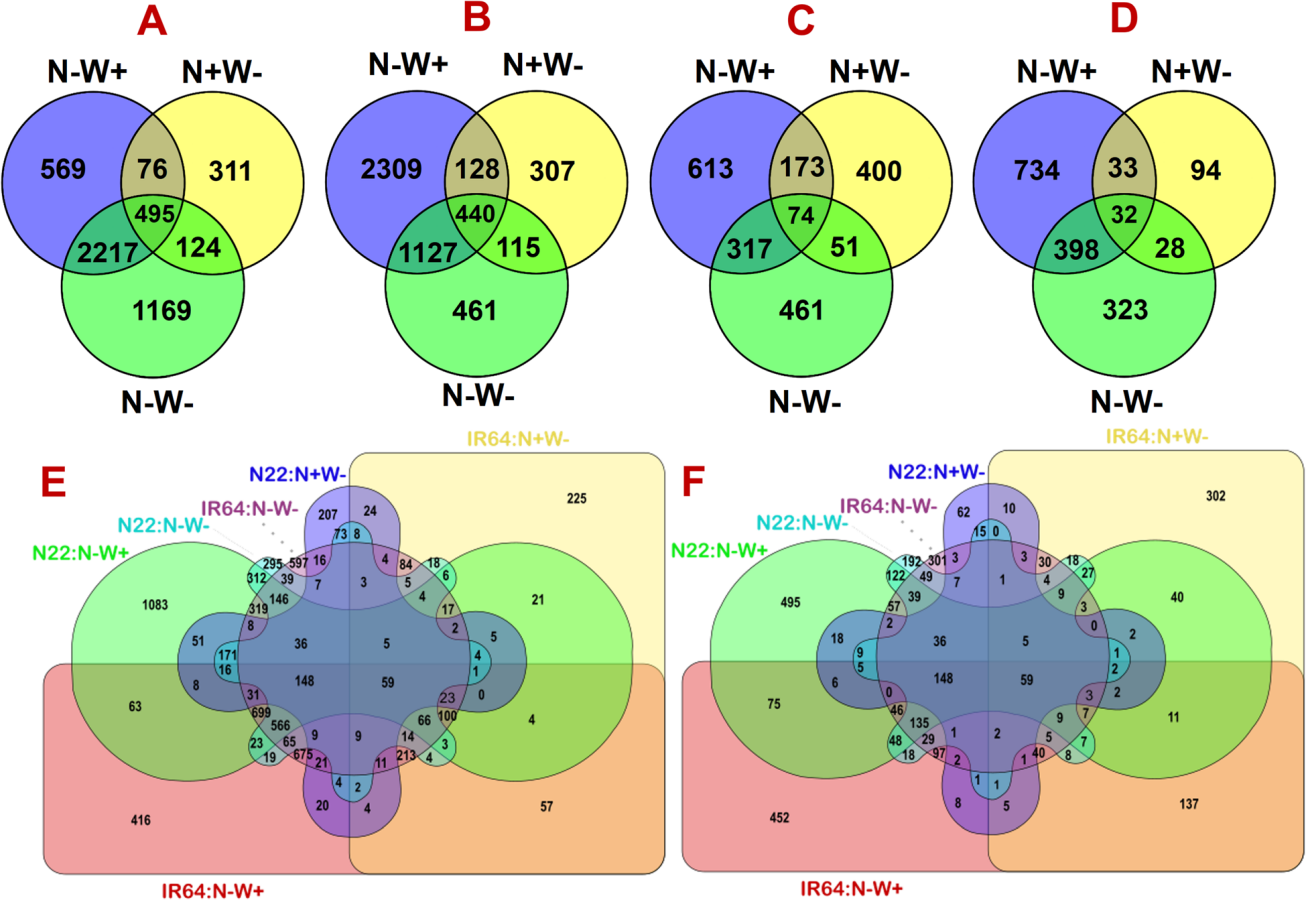
Comparison of common and unique DEGs identified across low N (N-W+), low water (N + W-) and dual stress (N-W-) treatments compared to optimal (N + W+) conditions in root (R) and shoot (S) tissues of two rice genotypes, IR64 and N22. **a**: DEGs in shoot tissues of IR64 across treatments; **b**: DEGs in shoot tissues of N22 across treatments; **c**: DEGs in root tissues of IR64 across treatments; **d**: DEGs in root tissues of N22 across treatments; **e**: DEGs identified in shoot tissues of both the genotypes; **f**: DEGs identified in root tissues of both the genotypes

We identified a total of 8926 unique differentially expressed genes (DEGs) across all the stress treatments by using N+W+ treatment as control for each stress (Supplementary Table 1). In brief, we identified only 1174, 698 and 903 DEGs in the root tissues of IR64, and 1197, 187 and 781 DEGs in N22; whereas nearly double the number of DEGs were found in the shoot tissues, i.e., 3357, 1006 and 4005 in IR64, and 4004, 990 and 2143 DEGs in N22 under N-W+, N+W- and N-W- stress treatments. Further, in the shoots of IR64, 2712 (67.7%) of the 4004 DEGs identified under the dual stress were common with N-W+ stress, while only 619 (15.46%) were common with N+W- stress (Fig. 8a). Similarly, in N22 shoots also, 1567 (73.12%) and 555 (25.9%) of the dual stress DEGs were common with N-W+ and N+W- stress (Fig. 8b). In root tissues also, the same trend was observed albeit in a lower proportion with 43.39% and 13.87% in IR64, and 55.06% and 7.68% DEGs in N22 from the N-W+ and N+W- stress DEGs were common with the dual stress DEGs (Figs. 8c and d). Overall, in IR64 shoots, only 23.6% (1169), 11.5% (559) and 6.3% (311) of the genes were unique to dual, N-W+ and N+W- stress respectively, whereas in N22, 9.4% (N-W-), 47.2% (N-W+), and 6.3% (N+W-) of the DEGs were unique. In case of the root tissues, 22.1%, 29.3% and 19.1% of DEGs were unique to dual, N- and W- stress in IR64, whereas 19.7% (N-W-), 44.7% (N-) and 5.7% (W-) of the DEGs were unique in N22. Thus, both in the root and shoot tissues of N22, more number of unique DEGs were identified under N- stress. Further, comparison across the genotypes revealed that just 11.7% (416/3353 of the DEGs in IR 64 under N-) to 27.08% (1083/4000 of the DEGs in N22 under N-) DEGs in shoot tissue were unique to a specific treatment and genotype, while the rest of the DEGs were common to one or more treatments or genotypes (Fig. 8e). However, in case of the root tissue, comparatively, a higher proportion of the DEGs were found to be unique to a specific treatment, which varied from 24.65% (192/779 of the DEGs in N22 under N-W-) to 43.27% (302/698 of the DEGs in IR64 under W-) with an exception of just 3.3% of DEGs (30/900) in IR64 under N-W- treatment (Fig. 8f). The common DEGs across the genotypes and treatments also showed a similar direction of regulation (either uniformly up or down) in both the genotypes (Supplementary Table 1).

### Comparison of the Expression of N Transporters, Sensors and Regulators under Various Stress Treatments in IR64 and N22

Of the 15 differentially expressed N transporter genes including the high affinity nitrate transporters, ammonium transporters and urea transporters, root tissues of IR64 showed differential expression for all of them, whereas N22 roots showed differential expression only in 11 under the N-W+ and/or N-W- stresses (Supplementary Table 2). Four N transporters and their accessory protein genes (*NRT2.1*, *NRT2.4*, *OsNAR2.2* and *OsAMT2_1*) were differentially expressed also under N+W- condition. A low affinity nitrate transporter, *OsNPF7.2*, was differentially expressed only in N22 shoots under all the three stress conditions implicating its genotype specific role in nitrate transport in the above ground part. Among these transporters a few more genes annotated as ‘similar to nitrate transporters’ (*Os10g0111300*, *Os10g0370700* and *Os06g0581000*) and others annotated as ‘similar to nitrate and chloride transporter’ (*Os03g0682100*) showed downregulation only in N22 shoot under N-W+ stress. Three negative regulators of N stress, i.e., *NIGT1*, *OsBT* and *OsACTPK1*, were downregulated in IR64 in either root or shoot tissues under both N-W+ and dual (N-W-) stress, while NIGT1 transcript alone was downregulated in N22 under N-W+ and dual stress. *OsACTPK1* was downregulated only under N-W+ stress in N22 roots, but not dual stress. *OsBT* was not downregulated in N22 under any stress in any of the tissues. More interestingly, NIGT1 transcript was found to be upregulated in N22 under N+W- stress. *Os04g0680400* and *Os12g0503000* encoding for allantoinase and allantoin transporter were downregulated under N-W+ and dual stress in both the genotypes but not under N+W- stress. Further, *Os02g0673100* encoding for aluminium induced malate transporter was found to be upregulated in both the root and shoot tissues of N22 and IR64 under N-W+ and dual stress, while aluminium induced citrate transporter encoded by *Os02g0673100* was upregulated only in IR64 roots under N+W- stress.

### Comparison of the Expression of Known Transcription Factors (TFs) and Novel (Not Annotated) Genes under Various Stress Treatments in IR64 and N22

A total of 173 transcription factors were found to be differentially expressed under different treatments (Supplementary Table 3), out of which 97 and 20 were specific to low nitrogen (N-W+) and low water (N+W-) supply respectively. The genotypic differences were also substantial in terms of the number of TFs expressed; 37 and 61 TFs were found to be expressed in N22 and IR64 respectively. The rest of the 75 DEGs showed a similar expression pattern in both the genotypes, except for two genes, namely, *Os01g0289732* and *Os03g0252900,* which encode a WRKY TF and a MYB like TF DIVARICATA, respectively. The former showed up- and down-regulated expression under N-W+ and N+W- stress in IR64 and N22 respectively, whereas the latter showed down- and up-regulated expression in IR64 and N22 respectively. Further, the expression pattern under N-W+ and dual stress were identical for most of the DEGs in IR64, while only a few DEGs showed such a pattern in N22. Of the 173 TFs, WRKY TFs were the most abundant with 30 genes followed by bZIP (18), MYB (17), HSF (13), AP2/ERF/AP1 (10), bHLH (9) and zinc finger (6) and NAC (6) genes. Of the zinc finger TFs, three were of Dof type, all of which were downregulated in N22 but not in IR64 under N-stress. A zinc finger, RING-type domain containing protein (*Os01g0123700*) was downregulated only in IR64 shoot tissues under low N (N-W+) but not in other tissues of IR64 or N22. *Os01g0213800*, encoding a plant specific transcription factor which is a target gene for mir319 was specifically upregulated (10.5 log_2_ fold change) in N22 shoots, only under N- stress. *Os08g0549600,* encoding a bZIP TF and *Os11g0523700* encoding a bHLH TF were the genes with higher folds of upregulation. The former was upregulated by 5–7 log_2_ fold in shoot tissues of both the genotypes under N-W+ and dual stress, while the latter was upregulated by 6.6 and 7.3 log_2_ fold in IR64 shoots under N-W+ and dual stress and 11.77 log_2_ fold in N22 shoots only under N-W+ stress. Another gene, *Os04g0659300*, encoding a receptor-like protein, which is implicated in root development, salt stress response and regulation of iron acquisition, was downregulated to 10 log_2_ fold change in the root tissues of both the genotypes suggesting the cross-talk of other nutrients with nitrogen.

Of the 8926 DEGs, 26.15% were completely novel, represented by 916 conserved hypothetical proteins and 1418 hypothetical genes/proteins. Of these transcripts, 20 hypothetical genes/proteins and 12 conserved hypothetical proteins had more than 8 log_2_ fold DE (Supplementary Table 4).

### Comparison of Genes Involved in Plant Hormone Metabolism and Receptors

Among the 44 DEGs involved in auxin biosynthesis and signalling, as many as 33 were shoot specific, out of which 26 DEGs were found to be specific to N-W+ or dual stress, whereas only 12 were specific to N+W- stress. Of the remaining 11 DEGs, only three were root specific while the others were expressed in both the tissues. As far as genotypic specificity is concerned, 15 and 12 DEGs were found to be specific to IR64 and N22 respectively (Supplementary Table 5). Similarly, in GA biosynthesis and signalling, 7 unique DEGs were found to be specific to N22 and IR64; 6 and 14 were root and shoot specific. *Os05g0227600* and *Os07g0418700* encoding proline rich glycoproteins with ABA dependent inhibition of root growth were downregulated only in IR64 roots under N-W+ or dual stress, whereas a similar gene, *Os05g0622900,* was upregulated in shoot tissues of N22 under both N-W+ and dual stress and in IR64 only under N-W+ stress. Further, *Os04g0511200,* an ABA responsive gene, was upregulated only in N22 under N+W- stress, but downregulated in shoot tissues of IR64 under N-W+ and dual stress. Another ABA dependent gene, *Os05g0381400,* differentially expressed only in N22 with upregulation under N+W- and downregulation under dual stress (Supplementary Table 5).

### GO Enrichment Analysis of DEGs across Stress Treatments and Genotypes

**Fig. 9:**
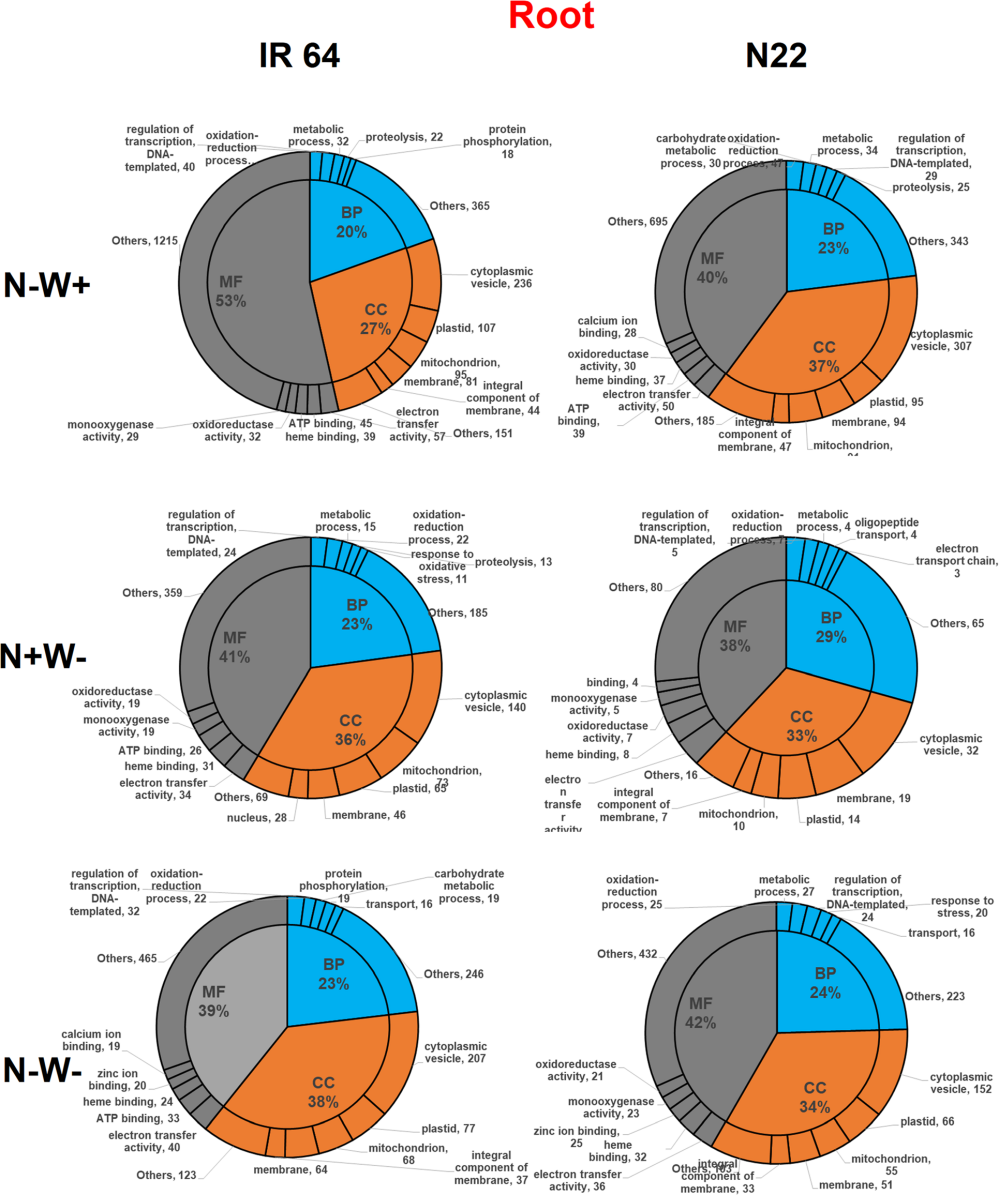
GO enrichment terms of the DEGs identified from various stress treatments across low N (N-W+), low water (N + W-) and dual stress (N-W-) treatments in root tissues of two rice genotypes, IR 64 and N22 divided into three major classes, i.e., biological processes (BP), cellular components (CC) and molecular function (MF) and most frequent subclasses

**Fig. 10:**
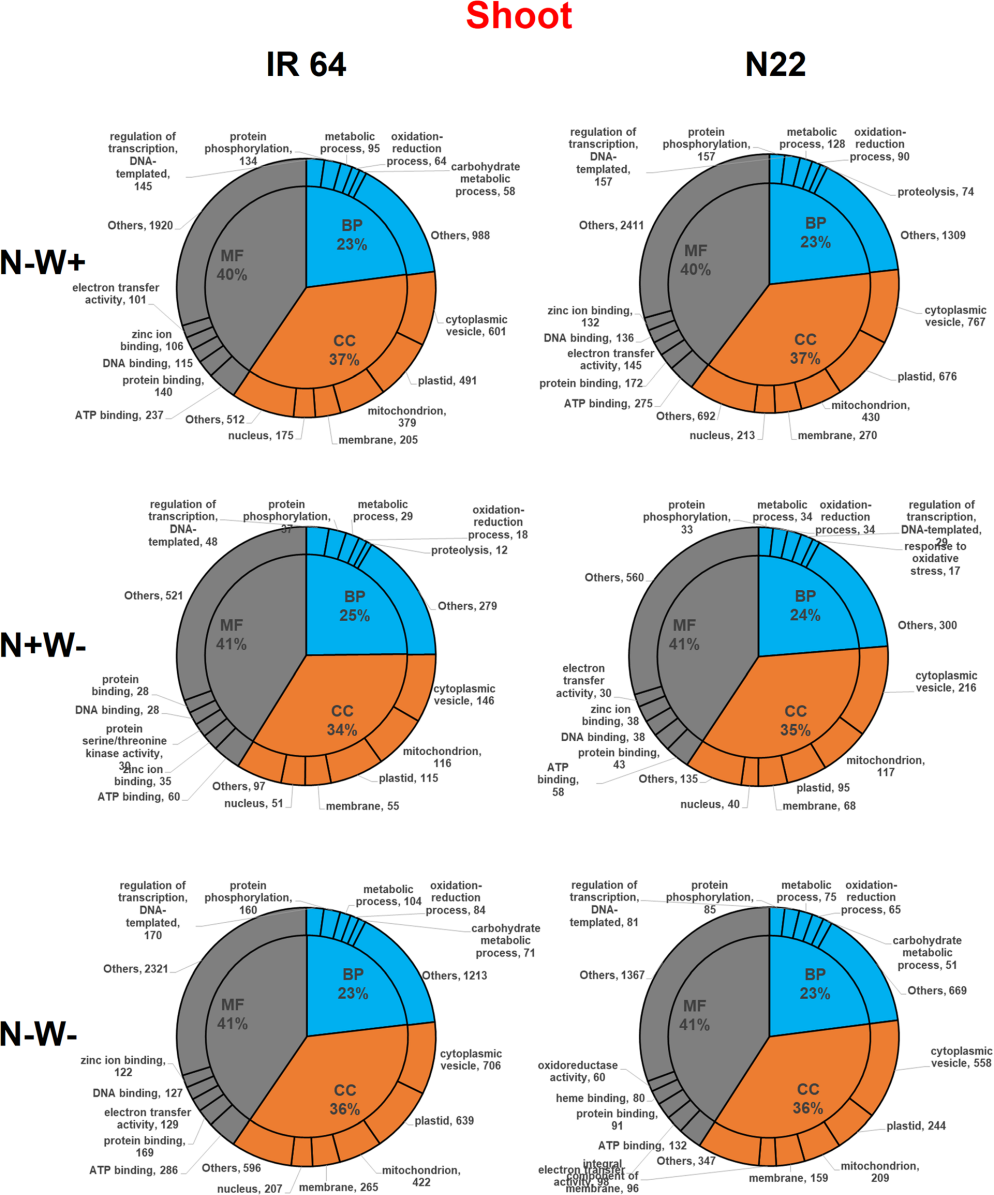
GO enrichment terms of the DEGs identified from various stress treatments across low N (N-W+), low water (N + W-) and dual stress (N-W-) treatments in shoot tissues of two rice genotypes, IR 64 and N22 divided into three major classes, i.e., biological processes (BP), cellular components (CC) and molecular function (MF) and most frequent subclasses

Across the genotypes and stress conditions, a similar proportion of the DEGs were classified into biological processes (22–25%), cellular components (34–37%) and molecular functions (40–41%) in shoot tissues of both the genotypes; however, in root tissues the response of genotypes varied under individual stresses (but similar in dual stress) with a huge difference in the proportion of genes (i.e., 53% and 40% in IR64 and N22 respectively) under molecular function (MF) category under N-W+ stress (Figs. 9 and 10). Further, sub-categorization of biological processes (BP) revealed that transcription factors (TFs) and genes involved in oxidation-reduction were equally abundant (40 genes in each category) in IR64 roots. Most of the DEGs belonged to the oxidation-reduction process (47) followed by metabolic processes (34), carbohydrate metabolism (30) and TFs (25) in N22 roots (Figs. 9). Under cellular components (CC), cytoplasmic vesicles were found to be the most important component and uniformly so across all treatments. Under W- stress in N22, membranes were the second most important components of CC. Under MF category, electron transfer activity followed by ATP, heme, and zinc binding protein were the key components in root tissues of both the genotypes.

IR64 under all the treatments and N22 under N-W+ condition showed a similar enrichment of genes under BP category in shoot tissues, with most of the DEGs falling under the subcategory of TFs, protein phosphorylation and metabolic processes. However, under N+W- stress, N22 showed an abundance of oxidative stress response genes too (Fig. 10). The GO enrichment pattern of genes in N22 shoots under N+W- and dual stress was similar for BP. Under CC, cytoplasmic vesicles were of the major category, while under MF, the ATP binding was the most important function in both the genotypes across treatments. The oxidation-reduction has been found to be the predominant process in the shoot tissues of N22 under MF category in dual stress conditions which distinctly differentiated N22 from IR64.

### Pathway Analysis of the DEGs

**Fig. 11:**
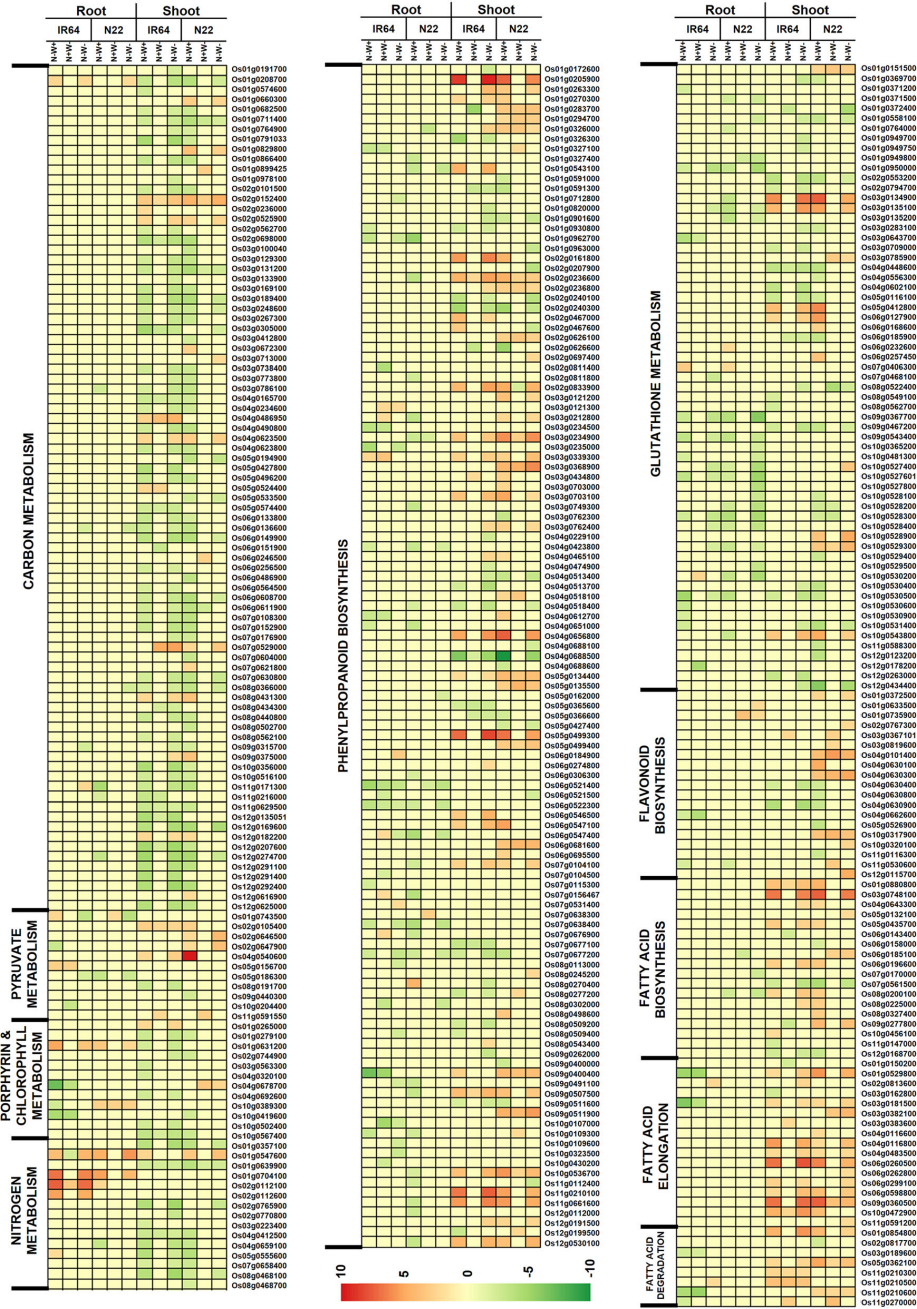
Heat map of the DEGs identified in some of the major metabolic pathways affected under N-W+, N-W- and N-W- stresses in root and shoot tissues of two rice genotypes, IR64 and N22

In order to gain a meaningful insight of the transcriptional control in the two genotypes under individual and dual stresses, we focused on specific pathways and genes identified from pathway analysis. Under the N-W+ condition, downregulation of a chloroplastic glutamine synthetase2, (GS2; *Os04g0659100*) involved in the reassimilation of the ammonia generated by photorespiration, was observed in N22 roots, but this was not the case in IR64 roots. Still, this gene was downregulated in the shoot tissues of both the genotypes under N-W+ conditions and IR64 under dual stress. *Os05g0555600* encoding amyloplastic glutamate synthase (GOGAT, similar to NADH-GOGAT) also showed the same pattern; it was downregulated in the shoot tissues of both the genotype under N-W+ and in IR64 under dual stress. Nitrogen metabolism was least affected under N+W- stress. However, in case of N transporters under dual stress, the IR64 roots showed upregulation for four of the N transporters while N22 showed upregulation only for two (Fig. 11). Both IR64 roots and shoots, and N22 roots (but not shoots) behaved similarly under the dual stress with as many as ten genes involved in N metabolism and transport downregulated (except for upregulation of a high affinity N transporter in roots). In N22 shoots, only as few as four genes were downregulated. Notably, *Os04g0659100*, *Os05g0555600* and *Os07g0658400* (Fd-GOGAT involved in N assimilation and leaf senescence) didn’t show downregulation in N22 shoots (Supplementary Table 2).

Comparison of carbon (C) metabolism including C-fixation by photosynthesis revealed that under N-W+ conditions, though similar sets of genes were downregulated in both the genotypes, a major difference was found in the differential upregulation of aspartate aminotransferase, encoded by *Os02g0236000,* in IR64 shoots, but not in N22 shoots (Fig. 11 and Supplementary Fig. 1). Under N+W- conditions, two genes, i.e., *Os01g0899425* and *Os02g0152400,* that encode the larger and smaller subunits of Rubisco respectively, were upregulated in N22 but only the latter was upregulated in IR64. Further, under N+W- stress, IR64 showed upregulation of genes (i.e., *Os07g0529000* and *Os04g0486950*) encoding isocitrate lyase and malate synthase known to be active in the glyoxylate pathway operating during germination. Pathway analysis suggested that under dual stress, the entire C_3_ carbon fixation cycle was downregulated in IR64 in addition to downregulation of regular TCA cycle and thus resulting in a comparatively lower pool of oxaloacetate in IR64 (Supplementary Fig. 1).

Both the genotypes did show upregulation of the gene encoding catalase (*Os03g0131200*), indicating the induction of ROS during these stresses, possibly resulting in minimization of hydrogen peroxide induced cell death. Flavonoid and phenylpropanoid biosynthesis processes were highly upregulated under N+W- stress and dual stress but not under N-W+ stress in the shoot tissue of N22 nor in IR64 shoot tissues under any of the three stresses (Fig. 11), implicating activation of secondary metabolite mediated stress management in the former genotype. Though fatty acid synthesis was upregulated under both N-W+ and dual stresses, it was downregulated under N+W- stress in both the genotypes, considerably higher fold in IR64 than that of N22. Further, the final steps in fatty acid elongation (supplementary Fig. 2) were found to be upregulated only in N22 under dual stress, while under N-stress it was downregulated; it did not show differential expression under W- stress. Genes involved in porphyrin and chlorophyll metabolism were completely downregulated in N22 under N- stress, but under W- and dual stress, it showed upregulation of the conversion process which formed chlorophyllide from divinyl chlorophyllide. This is also supported by our chlorophyll content measurements (Fig. 4). In IR64, the response to N- and dual stress was similar with respect to the downregulation of major chlorophyll synthesis pathway; still there was upregulation of conversion of bacterial geranyl geranyl chlorophyllide b and a to bacterial chlorophyll a and b **(**Fig. 11).

Photosynthesis was completely suppressed in IR64 shoots with the down regulation of 29, 3 and 31 genes under N-W+, N+W- and dual stress, while in N22 a large number of genes (Forster et al., 2007) were downregulated only under N-W+. Under N+W- stress, the chlorophyll a/b binding protein and photosystem II oxygen evolving complex PsbQ family protein encoded by *Os01g720500* and *Os02g0578400* were found to be upregulated while a photosystem II protein D1 was downregulated in N22 shoots. Under dual stress in N22, 2 genes each, namely, *Os02g0578400* and *Os04g0412200* (Ferredoxin I) were up and downregulated respectively (Fig. 11 and supplementary Fig. 3).

Glutathione metabolism in root and shoot tissues of both the genotypes showed substantial differential regulation especially under N-W+ and dual stress (Fig. 11 and supplementary Fig. 4). While root tissues of IR64 and N22 showed downregulation of 10 and 15 genes involved in glutathione metabolism under N-W+ stress, only 1 and 13 genes were found to be downregulated under dual stress. In the shoot tissues of IR64, only a fewer number of genes (4 up and 2 down) showed differential expression while in N22 as many as 19 genes showed differential expression (6 up and 13 down) under N-W+ stress. The scenario was quite distinct in case of dual stress with 14 genes in IR64 (4 up and 10 down) and 10 in N22 (6 up and 4 down) differentially expressed. We also noticed that the entire glutathione metabolism genes were physically located in a cluster on chromosomes 1 and 10 allowing common means for regulation (Supplementary Table 6).

Overall, in IR64 shoots, carbon fixation by photosynthesis was severely compromised and hence the feeding of pyruvate from glycolysis to cellular respiration was low under both N-W+ and dual stress conditions (Fig. 11 and supplementary Fig. 1). Still, pyruvate metabolism was partially upregulated, as seen from the higher activity of pyruvate kinase, in IR64 under both N-W+ and dual stress conditions (Figs. 7, 11 and supplementary Fig. 5), thereby creating a reasonable pool of acetyl CoA for further oxidative decarboxylation and subsequent ATP generation. However, under N+W- stress, the pyruvate metabolism was also comparatively more compromised with more accumulation of acetate and acetaldehyde rather than acetyl CoA. N22 showed poor response under N-W+ stress but a better response under N+W- stress as compared to IR64; the response under dual stress was completely different, with minimal impact on C fixation and comparatively higher pool of acetyl CoA. Specifically, genes (*Os01g0660300* and *Os03g0672300*) encoding pyruvate kinase were upregulated in N22 shoots under all the stress conditions, but downregulated (*Os11g0216000*) in IR64 shoots under N-W+ and dual stress (Fig. 11). Under N-W+ stress, in addition to glycolysis, pentose phosphate pathway was also compromised in both IR64 and N22 shoots but under dual stress, only IR64 showed downregulation and not N22.

### Validation of a Few Selected DEGs by qPCR Assay

**Fig. 12:**
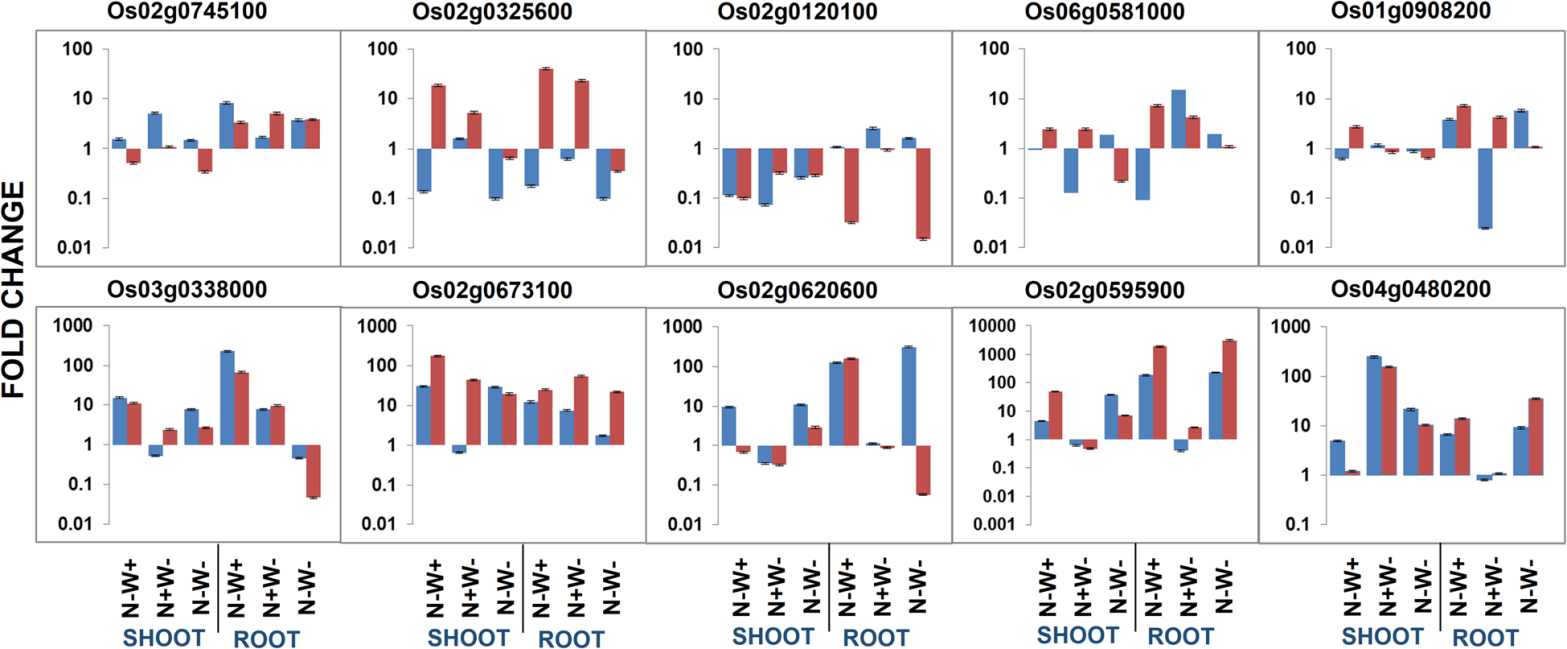
Validation of the selected differentially expressed genes using qPCR assay under N-W+, N + W- and dual stress (N-W-) conditions in IR64 and N22 rice genotypes

Primers for expression profiling of 10 DEGs were designed (Supplementary Table 7) to support the DEGs identified from the transcriptome data in the present study. Most of the genes selected for validation were involved in N transport and metabolism. The expression profiles of all the 10 selected genes obtained from qPCR assay matched with the pattern of differential expression observed in the transcriptome (Fig. 12 and supplementary Table 1). However, the fold change observed in the qPCR assay was higher than those obtained in the transcriptome for the nitrogen transporter genes. More importantly, the superiority of IR64 to chronic N-W+ stress through the transcriptional regulation of negative regulators of N transport (OsBT, NGT1 and OsACTPK1), high affinity nitrate transporters along with their accessory proteins, ammonium transporters (e.g., NRT1.2, NAR2.1, NAR2,.2 and AMT1_1) and N assimilation (AAT-glyoxylate) was well supported by the qPCR assay (Fig. 12 and supplementary Table 7).

### Identification of Low N Stress Specific QTLs

The yield data of the parents under N+ and N- field plots clearly demonstrated the contrasting nature of the parents for nitrogen use efficiency (Supplementary Fig. 6). Straw N levels were equal in the parental genotypes grown in both optimal and low N-plots (IR 64: 1.35 and 1.32% and N2: 1.085 and 1.09%) while grain N levels decreased in both the genotypes grown in low N- plots ((IR 64: 1.68 to 1.34%, N22: 1.40 to 1.02% for N+ to N- conditions). The mapping population of 253 individuals showed transgressive segregation for both the traits with a range of 0.77–3.47% and 0.67–1.62% for straw N and 0.4–2% and 0.04 to 1.9% for grain N under low and optimal N-plots respectively. Other than straw STI, all the traits showed a normal distribution (Supplementary Fig. 7). The coefficient of variation was nearly double for grain N (23.66% and 29.06% under N+ and N-) as compared to straw N (15.84% and 11.53% under N+ and N-). Thus, there was enough variation in the parents and the mapping population to attempt QTL mapping.

Though the mapping population was previously reported to be segregating for 1512 SNP markers, only 824 markers were utilized for construction of genetic linkage map and subsequent identification of heat tolerance QTLs by Shanmugavadivel et al. (2017). To improve the resolution of the map further, we relaxed the standards of goodness of fit for segregation distortion to *p* < 0.015 in the present study. Test for marker quality revealed that only one marker (SNP850) was the outlier, and hence it was removed from the analysis (supplementary Fig. 8). The genetic map consisted of 1262 markers with a map length of 1600 cM and 12 linkage groups.

For identification of QTLs, besides straw and grain N, when there is no external N supply (NN) and in the presence of external N (NP), STI and SSI of both these traits were also calculated. A total of 12 QTLs, one each for straw NN and seed STI, two for seed NP, three for seed NN and five for seed STI were identified on chromosomes 1, 5, 6 and 10 with LOD score greater than 3 (Table 2). Three of these 12 QTLs co-localized on chromosome 6 with their peak marker at 10.34–10.78 Mb for the traits seed NN and seed STI. Similarly, three more QTLs on chromosome 1 co-localized with their peak marker at 3.42–3.43 Mb for the traits seed NP, seed NN and seed STI. These QTLs explained phenotypic variation in the range of 4.9% to 22.3%. The QTL on chromosome 6 was the most robust with LOD score as high as 11 and R^2^ values as high as 0.177 to 0.223 for seed NN and seed STI. The favorable allele for this QTL was from IR64. In fact, all the favorable alleles were from IR64 for all the QTLs except for the one on chromosome 1 which governed seed NP, seed NN and seed STI.

**Table 2:** Details of the QTLs identified for straw and seed N content and their stress tolerance and susceptibility indices (STI and SSI) under no external N supply (NN) and optimal N (NP) supply in a recombinant inbred mapping population derived from IR64 and N22

Trait	Chromosome	Position (cM)	Physical Interval (Mb)		Size of the QTL region (Kb)	LOD	Additive effect	Phenotype variance explained (%)	Favourable QTL allele from
Straw_NN	10	86.21	16.94	17.71	769.74	3.72	0.05	6.7	IR64
Seed_NP	1	67.51	4.51	4.58	70.79	3.50	0.07	5.8	IR64
	1	342.51	37.37	37.88	513.10	4.07	−0.09	7.1	N22
Seed_NN	1	343.61	37.88	39.60	1719.29	3.43	−0.07	5.6	N22
	6	113.11	10.18	10.34	158.56	11.07	0.12	22.3	IR64
	6	118.71	10.96	11.02	66.35	8.61	0.11	17.7	IR64
Seed_SSI	6	167.51	19.66	20.38	715.09	4.20	−0.17	8.5	IR64
Seed_STI	1	51.11	3.46	3.50	41.10	3.05	0.08	4.9	IR64
	1	343.51	37.37	37.88	513.10	4.31	−0.11	7.1	N22
	6	113.11	10.18	10.34	158.56	10.30	0.17	20.6	IR64
	6	126.71	11.27	11.48	212.79	5.91	0.13	11.3	IR64
	4	295.71	34.36	34.41	52.97	4.54	0.14	7.7	IR64

### Co-Localization of the Major QTL with the Transcriptome Data of the Parental Genotypes under Optimal and Low N

**Fig. 13:**
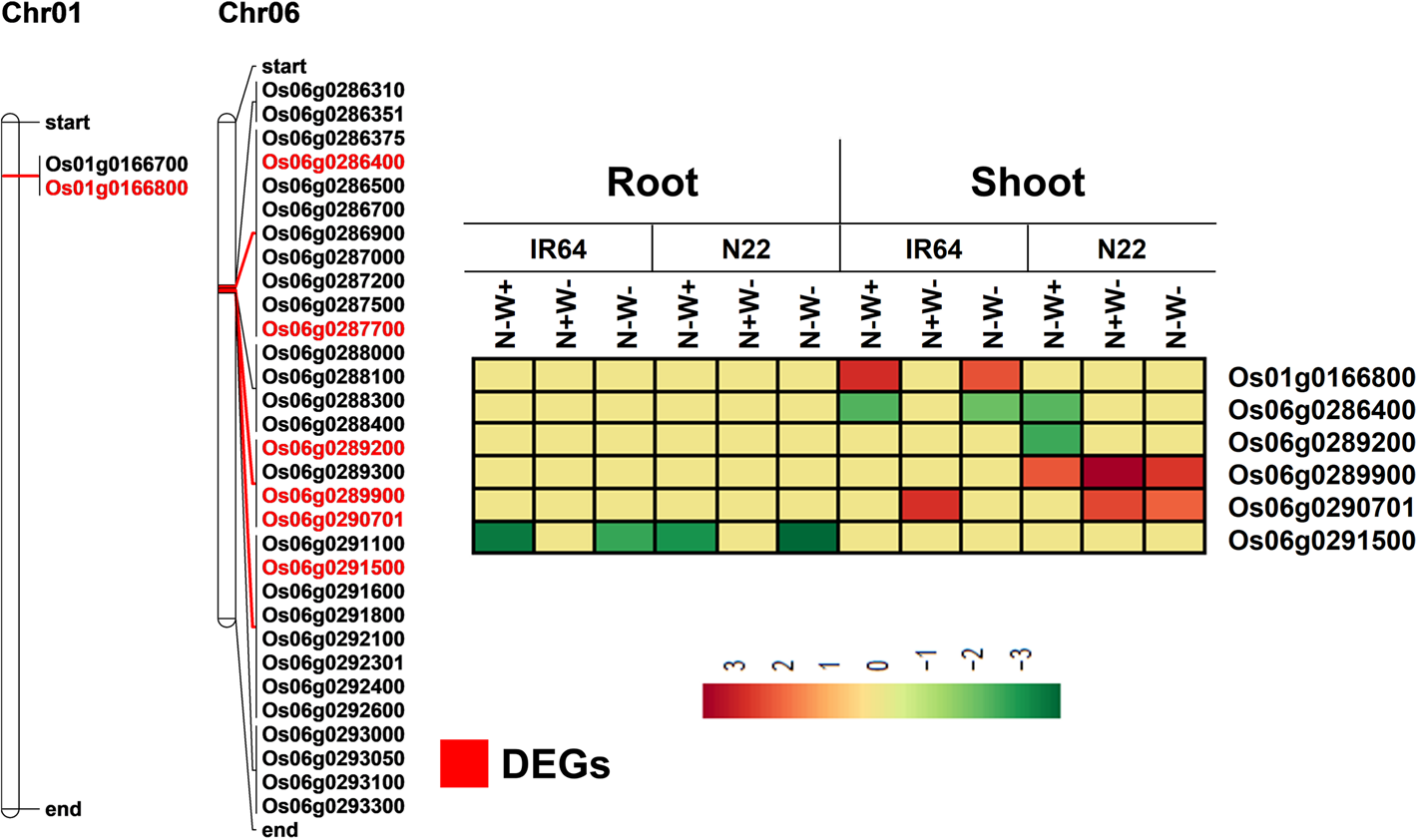
Comparison of expression profiles of the differentially expressed genes present in the two major QTL regions identified for straw and seed N content on chromosomes 1 and 6

We analyzed the two hot spot regions on chromosome 6 and chromosome 1 between the flanking SNP markers, and spanning a length of 439 Kbp and 10 Kbp respectively for the DEGs identified in the present study. The QTL hotspot on chromosome 6 comprised of 61 genes of which five were DEGs in the transcriptome analysis including a UDP-glucuronosyl/UDP-glucosyl transferase family protein (*Os06g289200*)*,* serine threonine kinase (*Os06g291500*)/ anthocyanidin 3-O-glucosyltransferase (*Os06g289900*)/ nitrate induced proteins (*Os06g286400*) and a hypothetical protein (*Os06g290701*). *Os06g289200* was downregulated only in N22 under N-W+ stress. Notably, the DEG involved in flavonoid metabolism (*Os06g289900*) was upregulated only in N22 under all the three stress conditions, more so under N+W- stress, reconfirming the robust antioxidant specific metabolism in N22. More importantly, serine threonine kinase (*Os06g291500*) was specific to N-stress as it was downregulated only under N-W+ and N-W- stresses but not under N+W- stress in both the genotypes. For all annotations, the latest MSU Rice IDs corresponding to the transcript IDs of the rap-db were used. Only for *Os06g290701*, no corresponding MSU ID was available. Blast analysis of the transcript sequence with the MSU pseudomolecule annotated it as a transposon (*LOC_Os06g18780*). In addition, there was a zinc finger Dof-type family protein in the vicinity which was downregulated in N22 under N-W+ stress but under both N-W+ and dual stress in IR64 (Fig. 13 and supplementary Table 1). The QTL on chromosome 1 harbored only two genes out of which *Os01g166800* encoding ETG1 (E2F TARGET GENE 1) protein showed differential expression only in IR64 shoots under N-W+ and dual stress.

### SNPs and Amino Acid Substitutions between IR64 and N22 in the Five Candidate Genes

We compared the CDS of the IR64 and N22 sequences for the five candidate genes using N22 sequences available in the database EMSgardeN22 (Sevanthi et al. 2018), the recent long read data generated to 66.8x coverage in the laboratory (unpublished) and the re-sequencing data of IR64 available in-house and at Rice-SNP seek database of IRRI (https://snp-seek.irri.org/). Of the 63 genes in the QTL hotspot regions, 56 had SNPs. The total number of SNPs across the 56 genes was 738 including the gene *LOC_Os06g291500* which encodes for serine threonine kinase and has two predicted isoforms. There were 6 and 3 non-synonymous (NS) amino acid substitutions in the two isoforms between IR64 and N22. Interestingly this gene (*Os06g0291500*) showed differential expression only in root tissues and under N-W+ and dual stress. The number of SNPs was the highest in *Os06g0289200* (28 SNPs) encoding for UDP-glucuronosyl/UDP-glucosyltransferase family protein) one of the five DEGs in the hotspot region, of which 11 resulted in NS amino acid substitutions (supplementary Table 8). One of the five candidate genes (*Os06g0286400*) encoding for a nitrate-induced NOI protein, had a single NS substitution. Further, another DEG, *LOC_Os06g18670* (*Os06g0289200*) encoding for anthocyanidin 3-O-glucosyltransferase, had 10 SNPs of which 3 resulted in NS substitutions. The large number of SNPs in other genes in the hotspot region mainly pertained to retroposon/transposon (like) elements which accounted for nearly one third of the genes i.e., 18 of the 56 (Supplementary Table 8). We also tried to look at the differences in the protein structure arising due to the 11 amino acid substitutions in case of *Os06g0289200* using I-TASSER which revealed similar tertiary structure of the protein between the two genotypes (supplementary Fig. 9). However, further simulations followed by stability analysis of the simulated structures are needed to arrive at a conclusion.

## Discussion

The enhanced root and limited shoot growth in rice under suboptimal water or nitrogen supply is well documented; this response is also known to be genotype specific with upland drought tolerant rice cultivars having deeper and more prolific rooting systems than lowland cultivars (Davidson, 1969; Steponkus et al., 1980, Dingkuhn and Kropff, 1996; Fageria and Moreira, 2011). Further, the extreme N-deficiency reduces branching and root hairs in cereals and legumes but increases the diameter of the lateral and first- and second-order nodal roots (Anderson et al. 1991). It is well known that the relative dry weight of rice roots decreased when N was omitted from a complete fertilizer (Baligar et al. 1998). Mild N-deprivation allows shoots to translocate photosynthates to root to modulate its structure in order to forage N from external media and this happens through modulation of brassinosteroid biosynthesis in Arabidopsis (Jia et al., 2020). However, if the stress is chronic/severe, growth of the root is restricted. Since chronic N stress was imposed in our study, we did observe a drastic reduction in the root dry weight, fresh weight and total root size (Figs. 2b, c and Figs. 5a) but not in root length (Figs. 2a and Figs. 5b) in both N-W+ and N-W- treatments. The increase in root length observed was also specific to IR64 under N-W+ stress. NUE efficient genotypes are known to improve the root system architecture in rice through a positive effect QTL, RDWN6XB (Anis et al., 2019). Further, through NRT1.1 transporter, the LRS is known to be curtailed under low soil nitrate levels in Arabidopsis (Tsay et al., 1993). Interestingly, such a modulation in RSA was not observed in the case in N+W- stress condition as it did not involve N- stress. Notably, the FOLRN and SOLRN values showed a clear genotype specific response with more reduction in N22 for FOLRN under single stresses (N-W+ and N+W-) but a drastic increase under dual stress (N-W-) conditions.

Since the RWC content did not vary substantially under N+W- condition, we repeated the entire experiment to measure the RWC content and the results are presented in Supplementary Table 9. We saw a clear pattern of RWC not significantly coming down under N+W- stress in this experiment too. Our results suggested that the effect of water stress was comparatively more pronounced in case of IR64 under N+W- conditions (RWC of 95.27% under N+W+ reduced to 86.36% under N+W-) compared to a minor change in N22 (89.37 to 84.67%). More interestingly, under the dual stress (N-W-), the RWC was similar to that of the N-W+ conditions, suggesting that lack of N somewhere compensated for lack of water too. In cotton, it was reported that stomatal behaviour might impart a stress avoidance type of drought resistance to N-deficient plants (Radin and Parker, 1979). This is despite the fact that water flux is important for nutrient flow in plants. Depending on the species, form of N supplied and dose of N, the N-water relations is reported to vary (Plett et al., 2020). This paper also describes the interactions of nitrate transporters and aquaporins with implications on root hydraulic conductivity. Further, K+ is also reported to have an impact on aquaporin expression. More the K+, lesser is the aquaporin-mediated N flow but with better WUE (Plett et al., 2020). In our data we can see down regulation of K transporters (mainly high affinity type) in shoot tissues of both the genotypes except for K transporter in the QTL region on chromosome 6 in N22 (Supplementary Table 6) as well as the K channel genes and K proton antiporter genes which were upregulated. In aquaporins also, we can see a lesser degree of downregulation in N-W- stress compared to N-W+ stress in both the genotypes (*Os02g0745100*, *Os03g0861300* and *Os07g0448400*).

Not only N- stress, but also water stress affects the transcriptional abundance of genes involved in N metabolism (Foyer et al. 1998; Singh and Ghosh, 2013). Reduced NR/NiR/GS/GOGAT activity is a sign of W- stress as reported in many crops including PEG treated *Malus prunifolia* which showed significantly lower NR activity though the NiR activity was not affected (Huang et al. 2011; Sánchez-Rodríguez et al., 2011). In rice, short-term (2–5 days) drought stress increased NiR but not NR activity (Pandey et al. 2010). All these results demonstrate that drought stress has a major influence on nitrate reduction and N assimilation which is supposed to be the main reason for the reduction of crop yield under drought stress (Wang et al. 2016). We observed that N-stress severely reduced NR activity in both the genotypes than that of water stress. More pronounced activity was observed in N22 than IR64 indicating efficient metabolism of N22 under water stress. NiR was found to have higher specific activity under N-stress and considerably so in IR64 than N22, potentially indicating more efficient N-assimilation in IR64.

Assimilation of inorganic ammonia to organic amino acid requires carbon skeleton from the TCA cycle, which is 2-oxoglutarate (2-OG). Therefore, N metabolism works in coordination with carbon (C) metabolism and hence, NUE is directly linked with both nitrogen as well as carbon metabolism in any crop (Sinha et al. 2018). Hence a drought resilient genotype is supposed to manage its N uptake and assimilation better under drought stress, besides the activity of C cycle enzymes, which we did find in N22 (Fig. 6). Notably, GS was found to be more active in IR64 and N22 under N- and W- conditions respectively, than that of the optimum condition; since GS is the first enzyme for NH_4_^+^ assimilation, these genotypes have a better mechanism to nullify the toxic effects of ammonium immediately after its release from various metabolic pathways (Masclaux-Daubresse et al. 2010). The drastic reduction of GOGAT under N-stress condition in both the genotypes indicates that no further transamination in chloroplast is preferred by either of these genotypes; possibly, glutamine itself is used as a N-transport molecule, or it transfers its amido group to aspartate in the presence of cytosolic asparagine synthatase in cytosol (Lam et al. 2003). In fact, alanine aminotransferase (AlaAT or ALT), asparagine synthase (AS), aspartate aminotransferase (AspAT), have all been implicated in modulating NUE (Shrawat et al. 2008; McAllister et al. 2016). We observed downregulation of *Osh36* (*Os05g0475400* encoding AAT like protein) in N22, and *ALT-2* in both the genotypes but more predominantly in N22. However, *Os03g0171900* and *Os03g0338900* encoding for proteins similar to *ALT-2* were upregulated only in N22. AspAT was also found to be downregulated in both the genotypes under dual stress. In case of IR64, down regulation of AspAT took place only under dual stress but not under N-stress.

Our observation based on N and C metabolic enzymes suggested better performance of IR64 and N22 under N- and W- conditions respectively (Fig. 6). We further looked at the DEGs to find whether they represent the differences in enzyme activities assayed in the study (supplementary Table 10). For five of the eight enzymes studies, namely, NR, NiR, PK, GS and GOGAT we could make two observations: i) the overall reduction in specific activity under N- stress and dual stress in both the genotypes were in correspondence with the downregulation of the respective DEGs; ii) the better performance of N22 under dual stress for PK and GS and IR64 for other enzymes was also supported by the DEGs. Further, we observed that not only N and C skeleton, but also high affinity N-transporter genes were influenced by W- stress with IR64 showing upregulation of *NRT2.1* (*Os02g0112100*) along with its accessory protein gene (NAR2.1) which is required for its activity as well as downregulation of NRT2.4 (*Os01g0547600*) in root tissues, possibly due to the lack of mass flow of nitrate to the root surface in the absence of water, and functional divergence of different nitrate transporters. Thus, IR64 clearly demonstrated better upregulation of overall N metabolism as well as N transporters under N- stress. We further observed that under N-(W+) stress, root fresh weight was higher in N22 while root dry weight was higher in IR64. This could be because of higher water status of a specific genotype. We hypothesize that though N22 is sensing the N- stress acutely it couldn’t do with much of its comparatively inefficient alleles of HATs. So, we suggest that N22 is trying to take up more water and forage N without any real growth and hence no subsequent addition to dry weight. However, the C-skeleton was better managed in N22 in N-, W- as well as dual stress, more so in the latter two stresses (Fig. 10; Sinha et al. 2018).

Both nitrogen and water use efficiency are very complex traits (Vikram et al. 2011; Wei et al. 2012; Feng et al. 2018; Sinha et al. 2018); yet, a large number of genes involved in N-metabolism with specific functions are known, e.g., high and low affinity N (nitrate/ammonium/urea) transporters, N-sensors, regulators of N uptake, N reduction and assimilation and also genes that promote efficient N use (such as *qNGR9* and *TOND1*), not only from the model plant *Arabidopsis* but also from rice, and consequently some of them have been exploited in breeding programs (Sun et al. 2014; Zhang et al., 2015). *Os12g0630100* encoding for thaumatin protein was found to be underlying the QTL, *TOND1*, by analyzing the fine mapped region of 279 kb using microarray expression data (Zhang et al. 2015). When we compared the DEGs present in the QTL region of *TOND1*, we found that there were five thaumatin or thaumatin like genes in that region, all of which showed differential expression under N- stress in IR64 shoots, three of them in IR64 root and two of them in N22 shoot (Supplementary Table 6). Some of them also showed differential expression in other stress (W- and dual) conditions in both the tissues. Further, only one of them, with an expected role in plant defense, was found to be upregulated in IR64 shoots while the remaining four were downregulated. Thaumatin proteins are mostly implicated in defense against bacterial and fungal pathogens or as allergens in plants (Datta et al., 1999, Wen et al. 2003; Kalpana et al. 2006; Liu et al. 2010). However, thaumatins have also been identified to be drought inducible and independent of ABA pathway in carrot (Jung et al. 2005). In this context, we suggest *TOND1* QTL to be a true one with not a single gene, but many genes showing similar function, governing the N-deficit response. We also found that both the genotypes tested in our study do not have favorable alleles in this region, in line with Zhang et al. (2015), wherein they reported that more than 70% of the rice cultivars did not have N-use efficient alleles for this locus, making it an attractive candidate for molecular breeding. On the other hand, we found upregulation of *DEP1* gene (*Os09g0441900*) underlying the QTL *qNGR9* in the shoots of both IR64 and N22 (Sun et al. 2014). Recently, *Os06g0270200* encoding for a potassium transporter-24 was found as the potential candidate gene for a major QTL (higher root dry weight under low N), i.e., qRDWN6^XB^, on chromosome 6 under N-deficiency in rice (Anis et al. 2019). We found upregulation of this gene in N22 shoots under drought stress but not under N- or dual stress. In fact, there were a total of 15 DEGs annotated as potassium transporters, of which 11 were downregulated and 4 were upregulated; interestingly all of them were specific to N- stress or dual stress except for *Os06g0270200* (Supplementary Tables 1 and 6), indicating the possibility of N and K cross-talk in rice.

From the major effect QTL regions identified in the present study, four potential candidate genes, encoding for UDP-glucuronosyl/UDP-glucosyltransferase, serine threonine kinase, anthocyanidin 3-O-glucosyltransferase, and nitrate induced proteins have been identified as they showed differential expression in N22 which also harbored 11, 6, 3 and 1 NS substitutions respectively between N22 and IR64. Interestingly the nitrate induced protein encoding gene is just 342 bp long which might have a regulatory role in N-stress response. Notably, this gene was not differentially regulated under water deficit stress in any of the genotypes (N+W-). No additional information is available as of now on this gene in the literature. *LOC_Os06g18670* is a known candidate gene in flavonoid metabolism and has been reported to be induced under various biotic and abiotic stresses and a known upstream gene for *Os07g0218700* encoding for a cytochrome P450 and is a major candidate genes for mQTLs in rice (Chen et al., 2018; Peng et al., 2020; Rice NetDB: http://bis.zju.edu.cn/ricenetdb/viewer/viewer.php?input=LOC_Os07g11870;). In our study flavonoid biosynthesis pathway was a major pathway differentially regulated under all the three stresses, especially in N22. UDP-glucosyltransferases (UGTs) are generally localized in the cytosol and involved in the biosynthesis of flavonoids, phenylpropanoids, terpenoids, and steroids, and also in the regulation of plant hormones (Lim et al. 2005; Liu et al. 2015). UGT glucosylation is implicated in the maintenance of ABA homeostasis in plants (Dong et al. 2014). Later, UGT71C5 was found to be the candidate gene that glycosylates ABA to ABA-GE (Liu et al. 2015). As *Os06g289200* harbored 11 NS substitutions and was specific to N- stress and downregulated only in N22 but not in IR64, the latter may have the favourable allele for this gene which helps in better ABA homeostasis and secondary metabolite-mediated N-stress in this genotype. Further, this gene is predicted to have four novel miRNA sequences (Supplementary Table 12), which need further experimental validation. Both the predicted isoforms of Serine threonine protein kinase encoding candidate gene (*Os06g0291500*) had several SNPs that lead to missense mutations. Though the function of this gene is not defined in rice, its homolog in Arabidopsis (At1g53660) is an organic anion transmembrane transporter and has been implicated in seed germination, development and biomass accumulation (Keurentjes et al., 2007; Hartanto et al., 2020). It may have a potential to cause differential NO_3_^−^ uptake in rice either directly or indirectly. *Os06g0291500* also had the presence of three known miRNAs namely miR1456, miR6252 and miR5487 (Supplementary Table 12). Further, the QTL hotspot regions also had four cadmium tolerance factor genes all of which had 1–5 missense mutations between the two genotypes under study. In *Brassica napus* the cadmium tolerance factor genes led to low NUE and vice-versa (Liao et al., 2019). However, these results should be interpreted with caution as the QTLs were identified in the fully grown plants on seed and straw N data while the transcriptome was from the seedling stage.

TFs are the major modulators of gene expression in plants. One of the popular TFs known to affect NUE, influencing N assimilation under low N-supply is *Dof* identified in maize (Yanagisawa et al. 2004), which was later shown to enhance NUE in a variety of crops including wheat and sorghum (Peña et al., 2017). A zinc finger Dof-type family protein found in the vicinity of the major QTL region identified on chromosome 6 in the present study, which was downregulated in N22 under N- stress but not in IR64, may be a potential candidate polymorphism for enhancing NUE in rice. Out of the 30 *Dof* TFs reported in rice (Lijavetzky et al., 2003), we found nine to be differentially expressed in our study, out of which only two expressed in the roots while the rest expressed in the shoots. Only one *Dof* (*Os10g0496000*) was found to be W- stress specific and was upregulated in the shoots of both the genotypes, while the rest eight were mostly specific to N- stress. Out of the eight, two were differentially expressed under dual stress in IR64 shoots. Further, except for the root-specific *Dof* gene (*Os07g0236700*) all were either upregulated in IR64 or downregulated in N22. However, we did not find a differential expression of Os04g0567800 transcript which was a known N-specific *Dof* gene in rice. Dof TFs are known to be regulating a variety of fundamental processes in plants, such as carbohydrate metabolism, photosynthetic carbon assimilation, vascular development and transport of sugar through phloem, phytochrome and light signaling, response to phytohormones and synthesis of storage proteins (Noguero et al., 2013). Among the other known TFs involved in NUE, *NTG1*, (NO_3_^−^-inducible and autorepressible transcriptional repressor) and *OsBT* (negative regulation of NO_3_^−^ uptake and NUE) were found to be downregulated in IR64 under both N- and dual stress while only the former was downregulated in N22. Further, in both the tissues, *OsACTPK1*, (regulator of N-uptake, the lack of which is known to enhance ammonium uptake) was found to be downregulated only in IR64 under both N- and dual stress (Supplementary Table 2; Beier et al., 2018), while it was downregulated in the root tissues of N22 under N- stress. Overexpression of *NGR5*, encoding for a AP2 domain TF, identified as a mutant (*ngr5*) with fewer tillers from genetic screens of semi-dwarf lines, has been recently shown to enhance NUE (Wu et al. 2020). This gene (*Os05g32270*) was found to be upregulated only in IR64 shoots both under N- and dual stress. Thus, there was a distinctly better transcriptional control of N-uptake mechanism operating in IR64 than N22, under N- as well as dual stress.

The differential expression of the genes under any stress or any other circumstances other than the optimal condition is routinely observed and documented. This is not because of the differences in the protein structure per se but possibly due to the differences in the promoter elements that control the expression. Hence, we attempted a cis-element analysis of 44 DEGs known to be implicated in N and water use efficiency by retrieving the 2 kb upstream sequences of these genes from the publically available resequencing data of IR64 and in-house available data of N22 (Sevanthi et al. 2018). We found differences in cis-motifs between the two genotypes in 25 of the 44 genes (of the differentially expressed transcripts) known to play a role in N metabolism (Supplementary Table 11). The cis-element variations in the DEGs including *thaumatin* and *Dof* gene and the five DEGs identified on chromosome 6 representing the known (*TOND1*) and novel QTLs provide a strong support to our findings in terms of DEGs as well as QTLs.

N22 is known for its efficient ROS scavenging activity which confer it with an effective layer of defence (tolerance) under drought stress; however, this was mainly explained through its efficient activity for SOD, catalase and peroxidase (Srivalli et al. 2003; Prakash et al. 2016). In our study, we observed another alternative mechanism of ROS scavenging derived from glutathione and flavonoid metabolism with more pronounced upregulation in N22 shoots not only under W- and dual stress but also under N- stress (Fig. 10).

Since the N- stress sensitive N22 genotype showed a mitigation of N- stress when W- stress was simultaneously applied, as compared to IR64, as seen from the multiple experiments in this study, we wanted to know whether ABA, a major pathway under drought/osmotic tolerance contributed to this. We did observe nine genes (Supplementary Table 13) which included 3 ABA related transcription factors, *Os04g0610400*, *Os05g0195200* and *Os06g0526600* encoding for an AP2 type TF, CCCH Zinc finger protein and DEAD box DNA helicase ATP binding protein and six genes which encoded for ABA dependent protein or ABA itself (*Os01g0963600*, *Os05g0226900*, *Os05g0227600*, *Os05g0381400*, *Os07g0418700* and *Os04g0511200*). Of these, *Os04g0511200* and *Os05g0227600* showed downregulation in IR64 under dual stress but not in N22. Again *Os05g0195200* (AP2 TF) showed upregulation only in N22 under dual stress but not in IR64. Since N22 is known for a better ABA dependent pathway as its drought stress response, the ABA receptors from N22 have been functionally shown to enhance the drought tolerance when overexpressed (Verma et al., 2019). However, *Os05g0226900* which is responsible for ABA dependent inhibition of root growth was found to be upregulated in both the genotypes. Some of the genes and miRNAs involved in ABA dependent pathway have shown to be exhibiting similar expression in drought resilient N22 as well as in drought sensitive genotypes like Pusa Basmati 1 (Rosetta et al., 2013; Sandhu et al., 2017). In the former, miR408 was shown be effective through its interaction with Ca-dependent proteins. To understand such mechanisms more work is needed so that the intricacies of the better dual stress of N22 is unraveled.

Allantoin is a key metabolite with a potential to improve N-homeostasis under stress. However, this metabolite was reported to have opposite fates in N- and W- stress in bread wheat, with significant accumulation under W- stress but enhanced catabolism under N- stress so as to have a better pool of ammonium ions for growth and survival (Casartelli et al. 2019). Our data also suggest significant catabolism of allantoin (upregulation of allantoinase) in both the genotypes under N- and dual stress. Recently, Redillas et al. (2019) have shown that overexpression of *OsUPS1* leads to allantoin accumulation under normal nitrogen supply while it promotes plant growth with better accumulation of allantoin in sink tissues under low N- supply. Lee et al. (2018) reported upregulation of *OsUPS1* under high N supply. In our transcriptome data, under both N- and dual stress, *OsUPS1* was highly downregulated in both the genotypes in root tissues. In shoot tissues, it was downregulated only under dual stress. Thus, it was clear that *OsUPS1* was responsible for non-accumulation of allantoin in tissues under N- stress. This response of allantoinase and *OsUPS1* was similar in both the genotypes. Though allantoin accumulation under drought stress has been reported by many workers (Watanabe et al., 2014; Plett et al. 2020) in a variety of crop plants, we did not find any change in the allantoin metabolism related genes under drought stress.

## Conclusion

The present study is the first report on genome-wide dual stress transcriptome analysis for low nitrogen and water stresses in rice using a pair of contrasting genotypes. The present study underpins the molecular basis underlying an efficient mechanism of nitrogen use metabolism operating in IR64 involving highly coordinated transcriptional regulation of negative regulators, various N transporters and key enzymes of N assimilation pathway. Though N22 has better N uptake and C metabolism, it has poor N assimilation and poor regulation of N transporters. N22 performed better under dual stress conditions owing to its better root architecture, chlorophyll and porphyrin synthesis and oxidative stress management through efficient glutathione metabolism and secondary metabolite production from flavonoid metabolism. The cis-element variations identified in some of the DEGs of the two contrasting genotypes supported the basis of the expression differences observed in the present study, more importantly the DEGs identified in the novel QTL regions. The transcriptome data can serve as a useful resource for further network analysis and characterization of key genes involved in low nitrogen and water stress in rice.

## Materials and Methods

### Plant Materials and Growth Conditions for Transcriptome Studies

Two rice genotypes, namely, IR 64 (IR64) and Nagina 22 (N22) adapted to irrigated-lowland and rainfed-upland ecosystems, respectively, were used in all pot-experiments in the present study. N22 is tolerant to drought stress but sensitive to low N supply, whereas IR64 is sensitive to drought stress but tolerant to low N supply (Mohapatra et al. 2014; Prakash et al. 2016; Sinha et al. 2018). Uniform sized seeds of IR64 and N22 were germinated and subsequently grown in pots of 4″ diameter containing perlite and vermiculite (1:2 (v/v) ratio) for 21 days following the method described earlier (Sinha et al. 2018)**.** Seedlings were grown in Yoshida medium under natural rice growing conditions, and subjected to three stress conditions, viz., low nitrogen (N-), low water (W-) and both low nitrogen and water (N-W-). N stress was applied to seedlings for the entire growth period, i.e., 21 days, whereas W stress was applied only during the 3rd week of the growth period (i.e., 15th-21st day). Drought stress was imposed for only 7 days as the leaves started showing complete rolling by then (Supplementary Fig. 1). NH_4_NO_3_ was used as the N source in the present study. It was supplied as a component of Yoshida medium at the rate of 8 mM (N+) and 0.08 mM (N-) for optimum and N- stress conditions respectively. To impose drought stress conditions, water from the nutrient medium was reduced to 1/5th of total volume keeping the concentration of N constant during the entire 3rd week of growth period. Thus, 100 ml of nutrient solution was used for ‘N’ experiments (both N+ and N-) while only 20 ml was used for drought stress treatment (W-). The nutrient media was given on every third day in order to maintain the availability of N and water as per the treatment i.e., the pots under dual stress received 0.08 mM of N supplied through 20 ml water on every third day in the third week while all other treatments received 100 ml of nutrient medium with the appropriate N concentrations. For each treatment, five pots containing five plants per pot were maintained. Biomass of the seedlings, root system architecture (RSA) traits and chlorophyll content were measured immediately after 21 days and remaining shoot tissues were kept in − 80 °C for assay of different N and C metabolizing enzymes and RNA isolation for transcriptome studies. The following symbols were used in this MS to indicate the different growth conditions: N+W+ (Both N and water are optimum), N-W+ (Low N stress but water optimum), N+W- (N optimum but low water stress) and N-W- (both low N and water stress).

### Plant Material and Growth Conditions for Genetic Mapping Studies on N Use Efficiency

A RI mapping population consisting of 281 RI lines derived by single seed descent method from a genetic cross of IR 64 X Nagina 22 was used for genetic mapping studies (Prakash et al. 2016; Shanmugavadivel et al. 2017). The mapping population along with the parents were grown in the N-depleted and N-optimal field plots of Indian Institute of Wheat and Barley Research, Karnal, India (29.7029° N, 76.9919° E) in *Kharif* 2016 (July-Oct), following the recommended agronomic practices in three rows of 1 m length with 15 cm X 10 cm spacing in order to map QTLs governing tolerance to low N supply. The nitrogen was supplied in the form of urea @ 100 Kg N ha^− 1^ (which is equivalent to 217 Kg of urea/ha) in an N-optimal plot while the N- treatment plot was not supplied with any N supplement. N-plot was developed by discontinuing external supply of any form of nitrogen since 2012, and maintained by growing Sorghum (*Sorghum bicolor*) for faster depletion of N. N content in the soil was measured from both the plots before the experiment. The available N in the N-optimal and N- plot was 150 and 90 Kg/ha respectively. Zero applied nitrogen field (a native field with no external N application in the specific season) has been reported to have 102 mg Nitrogen Kg^-1^ of soil (Srikanth et al., 2016; Rao et al. 2018). If we extrapolate this, considering average soil weight as 2200 t/ha, the available nitrogen in zero applied filed is around 224 Kg/ha. Hence our N- plot had considerably reduced nitrogen (90 Kg/ha) (it doesn’t reduce below this level mainly due to rain as well as irrigation water) compared to the N+ plot, where fertilizers are applied and regular crops are grown every year, which had 150 Kg N/ha. Further, in the N+ plot, the standard dose of 120 Kg/ha is applied. Hence the effective difference between N+ and N- field is 180 Kg of N/ha. However, the other soil physical and chemical properties are similar in both the plots which had Sandy Loam soil (sand 63.2%, silt 26.6%, clay 10.2%) with pH: 7.3; EC: 0.23 dSm^− 1^; OC: 0.42%; available P: 18.2 kgha^− 1^; available K: 232 kgha^− 1^; bulk density: 1.63 kgm^− 3^; FC: 18.9% and PWP: 7.3%. Of the 281 RILs, 253 showed good establishment in both the plots and hence were used in the mapping studies.

### Measurements of Morpho-Physiological Traits and Enzyme Activity in the Parental Genotypes, N22 and IR64

After 21 days of growth period, three seedlings from each pot were taken for the measurements of biomass, RSA traits and for enzyme analysis as per the methods described earlier, Sinha et al. (2015 and 2018). Length, fresh weight, and dry weight of shoot and root of properly washed seedlings were measured. For dry weight measurement, samples were dried at 50 °C till they attained constant weight. Chlorophyll was extracted in dimethyl sulfoxide (DMSO) and the content was measured according to the method described by Hiscox and Isarelstam (1965). Relative Water Content (RWC) of the leaves was measured using the following formula:

RWC (%) = [(W-DW) / (TW-DW)] × 100, where W, TW and DW represent fresh weight, turgid weight and dry weight of the samples, respectively (Barr and Weatherly, 1962).

RSA parameters such as the total root size (TRS; sum of path length of seminal and lateral roots), lateral root size (LRS; sum of path length of lateral roots as fraction of TRS), first-order LR number (FOLRN; number of lateral roots emerging from all the seminal roots), and second-order LR number (SOLRN; number of lateral roots emerging from first-order lateral root) were calculated from the data retrieved from the images of roots captured using a flatbed root scanner (Epson Perfection v700 Photo-Dual lens system, Seiko Epson Corporation, Nagano, Japan) at 400 dpi, and was analysed using the WinRhizo software (Regent Instruments Canada Inc., Arsenault et al. 1995).

The carbon (C) and nitrogen (N) metabolizing enzymes, viz., pyruvate kinase (PK), citrate synthase (CS) and nitrate reductase (NR), nitrite reductase (NiR), glutamine synthetase (GS), glutamate dehydrogenase (GDH), glutamate-oxoglutarate aminotransferase (GOGAT), were assayed in 200 mg of leaf tissues of rice seedlings as per the methods described in Sinha et al. (2015 and 2018). The specific activity of NR, NiR and GS was expressed as μmoles/mg/min, whereas the specific activities of GOGAT, GDH, PK, ICDH and CS were expressed as ∆OD/mg/min.

### RNA Extraction, RNA-Sequencing Using Next Generation Sequencing (NGS) Platform and Quantitative Real-Time PCR Assay

Two plants from each pot per treatment were sampled for transcriptome studies; they were thoroughly washed with sterile ddH_2_O and the root and shoot tissues were quickly separated and immediately frozen in liquid N. Total RNA was extracted from these tissues using the RNeasy Plant Mini Kit (Qiagen, India). RNA quality was assessed using the RNA 6000 Nano assay kit in the Bioanalyzer 2100 (Agilent, CA, USA). RNA samples from different biological replications were pooled, based on equimolar concentration, before library construction. A total of 16 libraries were constructed across the four treatments involving two tissues (root and shoot) and two genotypes (IR64 and N22) using the Truseq RNA sample preparation kit (Illumina, Singapore) following the manufacturer’s instructions. The insert size was 150 bp. The libraries were sequenced using paired end (2X100 bp) Illumina (Hiseq™ 2500) sequencing technology. The raw reads were submitted to NCBI and are available under the accession number GSE147158.

One μg of total RNA, isolated from individual samples was used to prepare cDNA using the Applied Bio-systems High-Capacity cDNA Reverse Transcription Kit (USA) as per the manufacturer’s instructions Quantitative real-time PCR (qRT-PCR) was performed for validation of some of the DEGs identified from the RNA-seq data. Reaction mixture of qRT-PCR was prepared using the required amount of diluted cDNA as template, 0.3 μl (10 picomole) of each primer, 15 μl 2xSYBR Green Master Mix (Agilent Technologies, USA) and 0.4 μl ROX fluorescence dye (diluted as per the instructions given in manual) and nuclease-free water for making a total volume of 30 μl qRT-PCR reaction mix. The thermal profile was as follows: 95 °C for 30 s, 60 °C for 15 s, and 72 °C for 20 s for 40 cycles of amplification. Three biological and technical replicates were used for the experiment including 18S gene which was used as a housekeeping gene for normalization.

### Phenotyping and Genotyping of the Mapping Population

Five plants each from the parents and 253 RILs grown in the progeny rows were randomly sampled from N+ and N- treatments and phenotyped. Panicles were separated from the rest of the plant and dried. The straw was finely chopped using a motorized chopper and then 20 g straw from each of the five plants was weighed, pooled, mixed homogenously and used for N estimation. Similarly, the grains were also ground and the entire sample of the five plants was pooled and mixed well for N estimation. Straw N and grain N were estimated in three replications from the pooled samples of each RIL using Dumas Nitrogen Analyzer NDA 701 (VELP Scientifica SRL, Italy) using 30–100 mg of sample. The N content was expressed both as % and mg g^− 1^. Besides straw N (NN for N- and NP for N+) and grain N (NN for N- and NP for N+) under N- and N+ conditions, four more traits, viz., stress tolerance index (STI) of straw and seed (Fernandez 1992) and stress susceptibility index (SSI) of straw and seed N content (Fischer and Maurer, 1978) were also calculated. In brief, STI was calculated as follows: STI = (N_P_ × N_N_)/(X_P_)^2^ where N_P_ is the N content of a genotype under optimal N supply; N_N_ is the N content under stress or no external N supply; and X_P_ is the mean N content of all genotypes under optimal N supply. Similarly, SSI was calculated as follows: SSI = 1-(N_N_/N_P_)/SI where, N_N_ and N_P_ were as in the case of STI while SI was calculated as: 1-(X_N_/X_P_) where X_N_ is the mean N content over genotypes under stress and X_P_ is the mean N content under optimal N supply over genotypes.

For DNA marker genotyping, the SNP genotyping data generated earlier on the same experimental material i.e., the RILs of IR64 X N22, for identification of heat tolerance QTLs, by Shanmugavadivel et al. (2017) was used. In brief, a customized 5K SNP array comprising 5246 SNPs, designed using the Illumina Infinium® II probes and dual colour channels (Kumar et al. 2015) was used for genotyping the parents, IR64 and N22 and the complete set of RILs.

### Data Analysis

All the morpho-physiological and enzyme based traits were analysed as described elsewhere (Sinha et al. 2018). In brief, all measurements were with three biological replicates and three technical replicates. Mean values were presented for all the parameters with error bar (standard error of means). F-test was conducted for comparison of means using ANOVA. Least significant difference (LSD) based critical difference (CD) at 5% was calculated for treatment × genotype interactions. All the analysis was done in MSTATC software. Curated SNP data of the mapping population was readily available from a previous study (Shanmugavadivel et al. 2017). For the transcriptome analysis, high quality reads were filtered from the raw reads by removing the low-quality reads (with Phred Score < 30 and read length < 36 bp) from 3′ and 5′ ends by the sliding window approach using sickle trimming tool [https://github.com/najoshi/sickle]. CLC genomics workbench v.12 was used for mapping the reads and identification of differentially expressed genes (DEGs). A rigorous comparison at FDR (False Discovery Ratio) *p* value < 0.05, and log_2_ fold change > 2 (for up regulation), > − 2 (for downregulation) was performed to select DEGs. For biologically meaningful comparisons, N+W+ treatment was used as a base (control) and DEGs were identified between N+W+ and each of the three stress treatments, viz., N-W+, N+W- and N-W-, for each tissue within a genotype. We previously reported the N responsive genes under optimal water supply, (i.e., N+W+ vs. N-W+) (Sinha et al. 2018); those were included again in the present study because only 47.35% of the reads could be mapped to the reference genome in the previous study as the parameter used for ‘mapping the reads’ was too stringent. Since CLC genomics workbench uses more flexible parameters of mismatch cost: 2, length fraction: 0.5 and similarity fraction: 0.8, more reads can be mapped. For the functional descriptions of the identified DEGs, the Rice Annotation Project Database (RAP-DB) was used. For further understanding of the behaviour of gene expression under nitrogen and drought stress, Gene Ontology (GO) terms enrichment analysis and pathway analysis of the DEGs were carried out using the agriGO (v2) web based tool (Gene Ontology Database Resource; geneontology.org) and the KEGG mapper.

Markers showing segregation distortion were excluded from the dataset prepared for map construction. Genetic linkage map was constructed using Kosambi function in MAPMAKER 3.0. Further, R/qtl package (Broman et al., 2003) was used to determine the quality of the genotyping data. Outliers in the genotype as well as phenotype datasets were removed. Composite interval mapping (CIM) was executed with 500 permutations in QTL cartographer for identification of QTLs for all the eight traits. Those loci detected with LOD score > 3 were declared as QTLs. The results of the QTL analysis and transcriptome studies were integrated by co-localizing the DEGs identified with the N specific QTLs identified in this study on the physical map of rice. Since we did not carry out QTL analysis under drought in the present study and the major QTLs known for drought stress are for ‘yield under drought stress’ (Vikram et al. 2015; Kumar et al. 2011), we did not attempt to integrate the drought specific DEGs with the drought QTLs.

## Supplementary Information

**Additional file 1: Supplementary Table 1**: All differentially expressed genes identified under N-W+, N + W- and N_W- treatments compared to optimal input supply (N + W+) in root and shoot tissues of IR64 and N22. **Supplementary Table 2**: Comparison of the expression of N transporters, sensors and regulators under various stress treatments in IR64 and N22. **Supplementary Table 3**: Comparison of the expression of known transcription factors (TFs) under various stress treatments in IR64 and N22. **Supplementary Table 4**: Comparison of the expression of novel (not annotated) genes under various stress treatments in IR64 and N22. **Supplementary Table 5**: Comparison of plant hormone metabolism genes and their receptors under various stress treatments in IR64 and N22. **Supplementary Table 6**: Comparison of the expression profile of the genes and QTLs known for Nitrogen use efficiency. **Supplementary Table 7**: Details of primers designed for validation of the DEGs identified from transcriptome analysis by qPCR assay. **Supplementary Table 8**: SNPs in the two major QTL hotspot regions identified on chromosomes 1 and 6. **Supplementary Table 9**: Relative water content (%) under optimal and all the three stress conditions measured in an independent experiment. **Supplementary Table 10**: DEGS identified in genes encoding for major enzymes involved in C and N skeleton. **Supplementary Table 11**: Details of variations in cis elements between IR64 and N22 genotypes for 20 N transporter, N regulator, TOND1, Dof genes and candidate genes identified from the major QTLs mapped in the present study. **Supplementary Table 12**: miRNAs in the candidate genes present in interval of the QTL hotspot region identified on chromosome 6. **Supplementary Table 13**: Comparison of expression of DE ABA related genes under different stress treatments in two rice genotypes N22 and IR64.

**Additional file 2: Supplementary Fig. 1**: Pathway analysis of glycolysis in shoot tissues under low nitrogen (N-), low water (W-) and dual stress (N-W-) in IR64 and N22rice genotypes.

**Additional file 3: Supplementary Fig. 2**: Pathway analysis of fatty acid metabolism in shoot tissues under low nitrogen (N-), low water (W-) and dual stress (N-W-) in IR64 and N22rice genotypes.

**Additional file 4: Supplementary Fig. 3**: Pathway analysis of photosynthesis in shoot tissues under low nitrogen (N-), low water (W-) and dual stress (N-W-) in IR64 and N22rice genotypes.

**Additional file 5: Supplementary Fig. 4**: Pathway analysis of glutathione metabolism in shoot tissues under low nitrogen (N-), low water (W-) and dual stress (N-W-) in IR64 and N22rice genotypes.

**Additional file 6: Supplementary Fig. 5**: Pathway analysis of pyruvate metabolism in shoot tissues under low nitrogen (N-), low water (W-) and dual stress (N-W-) in IR64 and N22rice genotypes.

**Additional file 7: Supplementary Fig. 6**: Yield of the two parental genotypes, IR 64 and Nagina 22 under N+ and N- plots.

**Additional file 8: Supplementary Fig. 7**: The frequency distribution of the eight traits based on straw and seed nitrogen content and their tolerant (STI) and susceptible (SSI) indices under no external N (NN) and optimal N (NP) supply.

**Additional file 9: Supplementary Fig. 8**: Quality assessment of polymorphic SNP markers between IR64 and N22 used for construction of genetic map and identification of QTLs in a recombinant inbred population. I-TASSER derived tertiary structure of the protein encoded by *Os06g0289200* in N22 and IR64 genotypes. There were 28 SNPs and 11 non-synonymous substitutions between the CDS and primary structure of the protein in this gene between IR64 and N22.

**Additional file 10: Supplementary 9**: I-TASSER derived tertiary structure of the proteins encoded by Os06g0289200 in N22 and IR64 genotypes. There were 28 SNPs and 11 non-synonymous substitutions between the CDS and primary structure of the protein in this gene between IR64 and N22.

## Data Availability

The complete datasets used in this study are presented in the manuscript and its supplementary material. The transcriptome data has been submitted to NCBI and are available under the accession number GSE147158. If any additional data generated during this study is sought they will be provided to the users.

## Abbreviations

QTL: Quantitative trait loci
DEG: Differentially expressed genes
NUE: Nitrogen use efficiency

## References

[CR1] Abid M, Tian Z, Ata-ul-karim ST, Cui Y, Liu Y (2016). Nitrogen nutrition improves the potential of wheat (Triticum aestivum L.) to alleviate the effects of drought stress during vegetative growth periods. Front Plant Sci.

[CR2] Anderson DS, Teyker RH, Rayburn AL (1991). Nitrogen form effects on early corn root morphological and anatomical development. J Plant Nutr.

[CR3] Anis GB, Zhang Y, Islam A, Zhang Y, Cao Y, Wu W (2019). RDWN6 XB , a major quantitative trait locus positively enhances root system architecture under nitrogen deficiency in rice. BMC Plant Biol.

[CR4] Baligar VC, Fageria NK, Elrashidi M (1998). Toxicity and nutrient constraints on root growth. Hort Sci.

[CR5] Barr HD, Weatherly PE (1962). A re-examination of the relative turgidity technique for estimating water deficit in leaves. Aust J Biol Sci.

[CR6] Beier MP, Obara M, Taniai A, Sawa Y, Ishizawa J, Yoshida H (2018). Lack of ACTPK1, an STY kinase, enhances ammonium uptake and use, and promotes growth of rice seedlings under sufficient external ammonium. Plant J.

[CR7] Broman KW, Wu H, Churchill GA (2003). R / QTL : QTL mapping in experimental crosses. Bioinformatics.

[CR8] Cai H, Lu Y, Xie W, Zhu T, Lian X (2012). Transcriptome response to nitrogen starvation in rice. J Biosci.

[CR9] Cao FY, Yoshioka K, Desveaux D (2011). The roles of ABA in plant-pathogen interactions. J Plant Res.

[CR10] Casartelli A, Melino VJ, Baumann U, Riboni M, Suchecki R, Jayasinghe NS (2019). Opposite fates of the purine metabolite allantoin under water and nitrogen limitations in bread wheat. Plant Mol Biol.

[CR11] Chen J, Qi T, Hu Z, Fan X, Zhu L, Iqbal MF, Yin X, Xu G, Fan X (2019). OsNAR2.1 positively regulates drought tolerance and grain yield under drought stress conditions in Rice. *Front*. Plant Sci.

[CR12] Chen J, Wang J, Chen W, Sun W, Peng M, Yuan Z, Shen S, Xie K, Jin C, Sun Y, Liu X, Fernie AR, Yu S, Luo J (2018). Metabolome analysis of multi-connected Biparental chromosome segment substitution line populations. Plant Physiol.

[CR13] Chen J, Zhang Y, Tan Y, Zhang M, Zhu L, Xu G (2016). Agronomic nitrogen-use efficiency of rice can be increased by driving OsNRT2.1 expression with the OsNAR2.1 promoter. Plant Biotechnol J.

[CR14] Dar M, Waza SA, Shukla S (2020). Drought tolerant Rice for ensuring food security in eastern India. Sustainability.

[CR15] Datta K, Velazhahan R, Oliva N, Ona I, Mew T, Khush GS (1999). Over-expression of the cloned rice thaumatin-like protein (PR-5) gene in transgenic rice plants enhances environmental friendly resistance to Rhizoctonia solani causing sheath blight disease. Theor Appl Genet.

[CR16] Davidson RL (1969). Effect of root / leaf temperature differentials on root / shoot ratios in some pasture grasses and clover. Ann Bot.

[CR17] Ding L, Li Y, Wang Y, Gao L, Wang M, Chaumont F (2016). Root ABA accumulation enhances Rice seedling drought tolerance under ammonium supply : interaction with Aquaporins. Front Plant Sci.

[CR18] Ding L, Lu Z, Gao L, Guo S, Shen Q (2018). Is nitrogen a key determinant of water transport and photosynthesis in higher plants upon drought stress?. Front Plant Sci.

[CR19] Dingkuhn M, Kropff M, Zamski E, Schaffer AA (1996). Rice. Photoassimilate distribution in plants and crops.

[CR20] Dong T, Xu ZY, Youngmin Park Y, Kim DH, Lee Y, Hwang I (2014). Abscisic acid uridine diphosphate glucosyltransferases play a crucial role in abscisic acid homeostasis in Arabidopsis. Plant Physiol.

[CR21] Fageria NK, A Moreira (2011) "The role of mineral nutrition on root growth of crop plants." *Advances in agronomy*. Vol. 110. Academic Press, 251–331

[CR22] Feng B, Chen K, Cui Y, Wu Z, Zheng T, Zhu Y (2018). Genetic dissection and simultaneous improvement of drought and low nitrogen tolerances by designed QTL pyramiding in rice. Front Plant Sci.

[CR23] Fernandez GCJ (1992). Effective selection criteria for assessing plant stress tolerance. In: Kus EG (ed) adaptation of food crop temperature and water stress. Proceeding of 4th international symposium.

[CR24] Fischer RA, Maurer R (1978). Drought resistance in spring wheat cultivars. I Grain yield responses. Aust J Agric Res.

[CR25] Forster P, Ramaswamy V, Artaxo P, Solomon S, Qin D, Manning M (2007). Changes in atmospheric constituents and in radiative forcing. Climate change 2007: the physical science basis. Contribution of working group I to the fourth assessment report of the intergovernmental panel on climate change.

[CR26] Foyer C, Valadier M, Migge A, Becker TW (1998). Nitrate Reductase activity and mRNA and the coordination of nitrogen and carbon metabolism.Full.Pdf. Plant Physiol.

[CR27] Gao Y, Li Y, Yang X, Li H (2010). Ammonium nutrition increases water absorption in rice seedlings (Oryza sativa L.) under water stress. Plant Soil.

[CR28] Guo S, Chen G, Zhou Y, Science A, Shen Q (2007). Ammonium nutrition increases photosynthesis rate under water stress at early development stage of rice (Oryza sativa L.). Plant Soil.

[CR29] Hartanto M, Joosen RVL, Snoek BL, Willems LAJ, Sterken MG, de Ridder D, Hilhorst HWM, Ligterink W, Nijveen H (2020). Network analysis prioritizes DEWAX and ICE1 as the candidate genes for major eQTL hotspots in seed germination of Arabidopsis thaliana. G3 (Bethesda).

[CR30] Hiscox JT, Israelstam GF (1965). A method for the extraction of chlorophyll from leaf tissue without maceration. Can J Bot.

[CR31] Jia Z, Giehl FHR, Wirén NV (2020). The root foraging response under low nitrogen depends on DWARF1 mediated brassinosteroid biosynthesis. Plant Physiol.

[CR32] Jung YC, Lee HJ, Yum SS, Soh WY, Cho DY, Auh CK, Lee TK, Soh HC, Kim YS, Lee SC (2005). Drought inducible but ABA independent thaumatin-like protein from carrot (Daucus carota L.). Plant Cell Rep.

[CR33] Kalpana K, Maruthasalam S, Rajesh T, Poovannan K, Kumar KK, Kokiladevi E (2006). Engineering sheath blight resistance in elite indica rice cultivars using genes encoding defense proteins. J Plant Sci.

[CR34] Keurentjes JJ, Fu J, Terpstra IR, Garcia JM, van den Ackerveken G, Snoek LB, Peeters AJ, Vreugdenhil D, Koornneef M, Jansen RC (2007). Regulatory network construction in Arabidopsis by using genome-wide gene expression quantitative trait loci. Proc Natl Acad Sci U S A.

[CR35] Kumar V, Singh A, Mithra SVA, Krishnamurthy SL, Parida SK, Jain S (2015). Genome-wide association mapping of salinity tolerance in rice (Oryza sativa). DNA Res.

[CR36] Lam H-M, Piu Wong, Hiu-Ki Chan, Kwan-Mei Yam, Li Chen, Cheung-Ming Chow, Gloria M. Coruzzi (2003). Overexpression of the ASN1 gene enhances nitrogen status in seeds of Arabidopsis. Plant Physiol 132 (2) 926–935, DOI: 10.1104/pp.103.020123 10.1104/pp.103.020123 PMC167031 12805621

[CR37] Lee DK, Redillas MCFR, Jung H, Choi S, Kim YS, Kim JK (2018). A nitrogen molecular sensing system, comprised of the ALLANTOINASE and UREIDE PERMEASE 1 genes, can be used to monitor N status in rice. Front Plant Sci.

[CR38] Leran S, Varala K, Boyer JC, Chiurazzi M, Crawford N, Daniel-Vedele F, David L, Dickstein R, Fernandez E, Forde B (2014). A unified nomenclature of NITRATE TRANSPORTER 1/PEPTIDE TRANSPORTER family members in plants. Trends Plant Sci.

[CR39] Li X, Ingvordsen CH, Weiss M, Rebetzke GJ, Condon AG, James RA (2019). Deeper roots associated with cooler canopies , higher normalized difference vegetation index , and greater yield in three wheat populations grown on stored soil water. J Expt Botany.

[CR40] Lian X, Wang S, Zhang J (2006). Expression profiles of 10,422 genes at early stage of low nitrogen stress in rice assayed using a cDNA microarray. Plant Mol Biol.

[CR41] Liao Q, Jian SF, Song HX, Guan CY, Lepo JE, Ismail AM, Zhang ZH (2019). Balance between nitrogen use efficiency and cadmium tolerance in Brassica napus and *Arabidopsis thaliana*. Plant Sci.

[CR42] Lijavetzky D, Carbonero P, Vicente-Carbajosa J (2003). Genome-wide comparative phylogenetic analysis of the rice and Arabidopsis Dof gene families. BMC Evol Biol.

[CR43] Lim E, Doucet CJ, Hou B, Jackson RG, Abrams SR, Bowles DJ (2005) Resolution of ( + ) -abscisic acid using an Arabidopsis glycosyltransferase. Tetrahedron Asymmetry 16(1):143–147. 10.1016/j.tetasy.2004.11.062

[CR44] Liu JJ, Sturrock R, Ekramoddoullah AKM (2010). The superfamily of thaumatin-like proteins: its origin, evolution, and expression towards biological function. Plant Cell Rep.

[CR45] Liu Z, Yan JP, Li DK, Luo Q, Yan Q, Liu Z. Bin (2015) UDP-glucosyltransferase71C5, a major glucosyltransferase, mediates abscisic acid homeostasis in Arabidopsis. Plant Physiol 167, 1659–1670. doi:10.1104/pp.15.00053, 4 10.1104/pp.15.00053 PMC4378179 25713337

[CR46] Loddo S, Gooding M (2012). Semi-dwarfing (Rht-B1b) improves nitrogen-use efficiency in wheat, but not at economically optimal levels of nitrogen availability. Cereal Res Commun.

[CR47] Lou D, Chen Z, Yu D, Yang X. (2020) SAPK2 contributes to rice yield by modulating nitrogen metabolic processes under reproductive stage drought stress. Rice (New York, N.Y.), 13(1), 35. https://doi.org/10.1186/s12284-020-00395-3 10.1186/s12284-020-00395-3 PMC7280414 32514747

[CR48] Ma K, Liu YG (2018). DELLA-GRF4-mediated coordination of growth and nitrogen metabolism paves the way for a new green revolution. Sci China Life Sci.

[CR49] Masclaux-Daubresse C, Daniel-Vedele F, Dechorgnat J, Chardon F, Gaufichon L, Suzuki A (2010). Nitrogen uptake, assimilation and remobilization in plants: challenges for sustainable and productive agriculture. Ann Bot.

[CR50] McAllister CH, Wolansky M, Good AG (2016). The impact on nitrogen-efficient phenotypes when aspartate aminotransferase is expressed tissue-specifically in Brassica napus. New Plant Sci.

[CR51] Mohapatra T, Robin S, Sarla N, Sheshashayee M, Singh AK, Singh K (2014). EMS induced mutants of upland rice variety Nagina22: generation and characterization. Proc Indian Natl Sci Acad.

[CR52] Noguero M, Atif RM, Ochatt S, Thompson RD (2013). The role of the DNA-binding one zinc finger (DOF) transcription factor family in plants. Plant Sci Int J Exp Plant Biol.

[CR53] Pandey A, Rajamani U, Verma J, Subba P, Chakraborty N, Datta K (2010). Identification of extracellular matrix proteins of rice (Oryza sativa L.) involved in dehydration-responsive network: a proteomic approach. J Proteome Res.

[CR54] Peleg Z, Blumwald E (2011). Hormone balance and abiotic stress tolerance in crop plants. Curr Opin Plant Biol.

[CR55] Peña PA, Quach T, Sato S, Ge Z, Nersesian N, Changa T (2017). Expression of the maize dof1 transcription factor in wheat and sorghum. Front Plant Sci.

[CR56] Peng Y, Zhang X, Liu Y, and Chen X (2020) Exploring heat-response mechanisms of MicroRNAs based on microarray data of Rice post-meiosis panicle Int J Genomics doi.org/10.1155/2020/7582612 10.1155/2020/7582612 PMC7519984 33015150

[CR57] Plett DC, Ranathunge K, Melino VJ, Kuya N, Uga Y, Kronzucker HJ (2020). The intersection of nitrogen nutrition and water use in plants: new paths toward improved crop productivity. J Exp Bot.

[CR58] Prakash C, Mithra SVA, Singh PK, Mohapatra T, Singh NK (2016). Unraveling the molecular basis of oxidative stress management in a drought tolerant rice genotype Nagina 22. BMC Genomics.

[CR59] Prasertsak A, Fukai S (1997). Nitrogen availability and water stress interaction on rice growth and yield. F Crop Res.

[CR60] Radin JW, Parker LL (1979). Water relations of cotton plants under nitrogen deficiency II. Environmental interactions on stomata. Plant Physiol.

[CR61] Rao, IS, Neeraja, CN, Srikanth B., Subrahmanyam D, Swamy KN, Rajesh K, Vijayalakshmi P, Kiran TV, Sailaja N, Revathi P, Rao PR, Rao LVS, Surekha K, Babu VR, Voleti SR (2018) Identification of rice landraces with promising yield and the associated genomic regions under low nitrogen. Sci Rep 8, 9200. https://doi.org/10.1038/s41598-018-27484-0, 1 10.1038/s41598-018-27484-0 PMC6003918 29907833

[CR62] Raun WR, Johnson GV (1999). Improving nitrogen use efficiency for cereal production. Agron J.

[CR63] Redillas MCFR, Bang SW, Lee DK, Kim YS, Jung H, Chung PJ (2019). Allantoin accumulation through overexpression of ureide permease1 improves rice growth under limited nitrogen conditions. Plant Biotechnol J.

[CR64] Rosetta M, Balyan SC, Kansal S, Agarwal P, Kumar S, Kumar M, Raghuvanshi S (2013). Evolution of variety specific regulatory scheme for expression of Osa-miR408 in indica rice varieties under drought stress. FEBS J.

[CR65] Sánchez-Rodríguez E, del Mar Rubio-Wilhelmi M, Ríos JJ, Blasco B, Rosales MÁ, Melgarejo R (2011). Ammonia production and assimilation: its importance as a tolerance mechanism during moderate water deficit in tomato plants. J Plant Physiol.

[CR66] Sandhu M, Sureshkumar V, Prakash C, Dixit R, Solanke AU, Sharma TR, Mohapatra T, S. V. AM (2017). RiceMetaSys for salt and drought stress responsive genes in rice: a web interface for crop improvement. BMC Bioinformatics.

[CR67] Sevanthi AMV, Kandwal P, Kale PB, Prakash C, Ramkumar MK, Yadav N, Mahato AK, Sureshkumar V, Behera M, Deshmukh RK, Jeyaparakash P, Kar MK, Manonmani S, Muthurajan R, Gopala KS, Neelamraju S, Sheshshayee MS, Swain P, Singh AK, Singh NK, Mohapatra T, Sharma RP (2018). Whole genome characterization of a few EMS-induced mutants of upland Rice variety Nagina 22 reveals a staggeringly high frequency of SNPs which show high phenotypic plasticity towards the wild-type. Front Plant Sci.

[CR68] Shanmugavadivel PS, Mithra AS, Prakash C, Ramkumar MK, Tiwari R, Mohapatra T (2017). High resolution mapping of QTLs for heat tolerance in rice using a 5K SNP array. Rice.

[CR69] Shrawat AK, Carroll RT, DePauw M, Taylor GJ, Good AG (2008). Genetic engineering of improved nitrogen use efficiency in rice by the tissue-specific expression of alanine aminotransferase. Plant Biotechnol J.

[CR70] Singh KK, Ghosh S (2013). Regulation of glutamine synthetase isoforms in two differentially drought-tolerant rice (Oryza sativa L.) cultivars under water deficit conditions. Plant Cell Rep.

[CR71] Sinha SK, Rani M, Bansal N, Gayatri VK, Mandal PK (2015). Nitrate starvation induced changes in root system architecture, carbon: nitrogen metabolism, and miRNA expression in nitrogen-responsive wheat genotypes. Appl Biochem Biotechnol.

[CR72] Sinha SK, Sevanthi AM, Chaudhary S, Punit T, Venkadesan SK (2018). Transcriptome analysis of two Rice varieties contrasting for nitrogen use efficiency under chronic N starvation reveals differences in chloroplast. Genes (MDPI).

[CR73] Sinha SK, Tyagi A, Mandal PK (2019). External nitrogen and carbon source mediated response on modulation of root system architecture and nitrate uptake in wheat seedlings. J Plant Growth Regul.

[CR74] Snyder CS, Bruulsema TW, Jensen TL, Fixen PE (2009). Review of greenhouse gas emissions from crop production systems and fertilizer management effects. Agric Ecosystems Environ.

[CR75] Srikanth B, Subhakara Rao I, Surekha K, Subrahmanyam D, Voleti SR, Neeraja CN (2016). Enhanced expression of OsSPL14 gene and its association with yield components in rice (Oryza sativa) under low nitrogen conditions. Gene.

[CR76] Srivalli B, Sharma G, Khanna-Chopra R (2003). Antioxidative defense system in an upland rice cultivar subjected to increasing intensity of water stress followed by recovery. Physiol Plant.

[CR77] Steponkus PJ, Cutler JM, O’Toole JC, Turner NC, Kramer PJ (1980). Adaptation to water stress in rice. Adaptation of plants to water and high temperature stress.

[CR78] Sun J, Bankston JR, Payandeh J, Hinds TR, Zagotta WN, Zheng N (2014). Crystal structure of the plant dual-affinity nitrate transporter NRT1.1. Nature.

[CR79] Tsay YF, Schroeder JI, Feldmann KA, Crawford NM (1993). The herbicide sensitivity gene Chl1 of Arabidopsis encodes a nitrate-inducible nitrate transporter. Cell.

[CR80] Verma RK, Santosh Kumar VV, Yadav SK, Pushkar S, Rao MV, Chinnusamy V (2019). Overexpression of ABA receptor *PYL10* gene confers drought and cold tolerance to Indica Rice. Front Plant Sci.

[CR81] Vikram P, Swamy BPM, Dixit S, Ahmed HU, Teresa M, Cruz S Singh AK, Arvind Kumar (2011) qDTY 1.1, a major QTL for rice grain yield under reproductive-stage drought stress with a consistent effect in multiple elite genetic backgrounds. BMC Genet, 12, 89. https://doi.org/10.1186/1471-2156-12-89 10.1186/1471-2156-12-89 PMC3234187 22008150

[CR82] Vikram P, Swamy BPM, Dixit S, Singh R, Singh BP, Miro B (2015). Drought susceptibility of modern rice varieties: an effect of linkage of drought tolerance with undesirable traits. Sci Rep.

[CR83] Wang M, Ding L, Gao L, Li Y, Shen Q, Guo S (2016) The interactions of Aquaporins and mineral nutrients in higher plants. Int J Mol Sci 17(8). 10.3390/ijms17081229 10.3390/ijms17081229 PMC5000627 27483251

[CR84] Wang M, Shen Q, Xu G, Guo S (2014) New insight into the strategy for nitrogen metabolism in plant cells. Int Rev Cell Mol Biol. 310:1–37. 10.1016/B978-0-12-800180-6.00001-3 10.1016/B978-0-12-800180-6.00001-3 24725423

[CR85] Watanabe S, Matsumoto M, Hakomori Y, Takagi H, Shimada H, Sakamoto A (2014). The purine metabolite allantoin enhances abiotic stress tolerance through synergistic activation of abscisic acid metabolism. Plant Cell Environ.

[CR86] Wei D, Cui K, Ye G, Pan J, Xiang J, Huang J (2012). QTL mapping for nitrogen-use efficiency and nitrogen-deficiency tolerance traits in rice. Plant Soil.

[CR87] Wen N, Chu Z, Wand S (2003). Three types of defense-responsive genes are involved in resistance to bacterial blight and fungal blast diseases in rice. Mol Gen Genomics.

[CR88] Wu K, Wang S, Song W (2020). Enhanced sustainable green revolution yield via nitrogen-responsive chromatin modulation in rice. Science.

[CR89] Xiong J, Li J, Wang H, Zhang C, Naeem MS (2018). SC. Plant Physiol Biochem.

[CR90] Xu G, Fan X, Miller AJ (2012). Plant nitrogen assimilation and use efficiency. Annu Rev Plant Biol.

[CR91] Xu K, Xu X, Fukao T, Canlas P, Maghirang-Rodriguez R, Heuer S (2006). Sub1A is an ethylene-response-factor-like gene that confers submergence tolerance to rice. Nature.

[CR92] Yanagisawa S, Akiyama A, Kisaka H, Uchimiya H, Miwa T (2004). Metabolic engineering with Dof1 transcription factor in plants: improved nitrogen assimilation and growth under low-nitrogen conditions. Proc Natl Acad Sci U S A.

[CR93] Yang H, Menz J, Ha I, Benz M, Fujiwara T, Ludewig U (2015). High and low affinity urea root uptake : involvement of NIP5;1. Plant Cell Physiol.

[CR94] Zeigler RS, Mohanty S (2010). Support for international agricultural research: current status and future challenges. New Biotechnol.

[CR95] Zhang Y, Tan L, Zhu Z, Yuan L, Xie D, Sun C (2015). TOND1 confers tolerance to nitrogen deficiency in rice. Plant J.

