# Supplementary figures and images for: Integration of Dual Stress Transcriptomes and Major QTLs from a Pair of Genotypes Contrasting for Drought and Chronic Nitrogen Starvation Identifies Key Stress Responsive Genes in Rice
Source: Rice (N Y). 2021 Jun 5;14:49. doi: 10.1186/s12284-021-00487-8 (PMC8179884; doi:10.1186/s12284-021-00487-8)

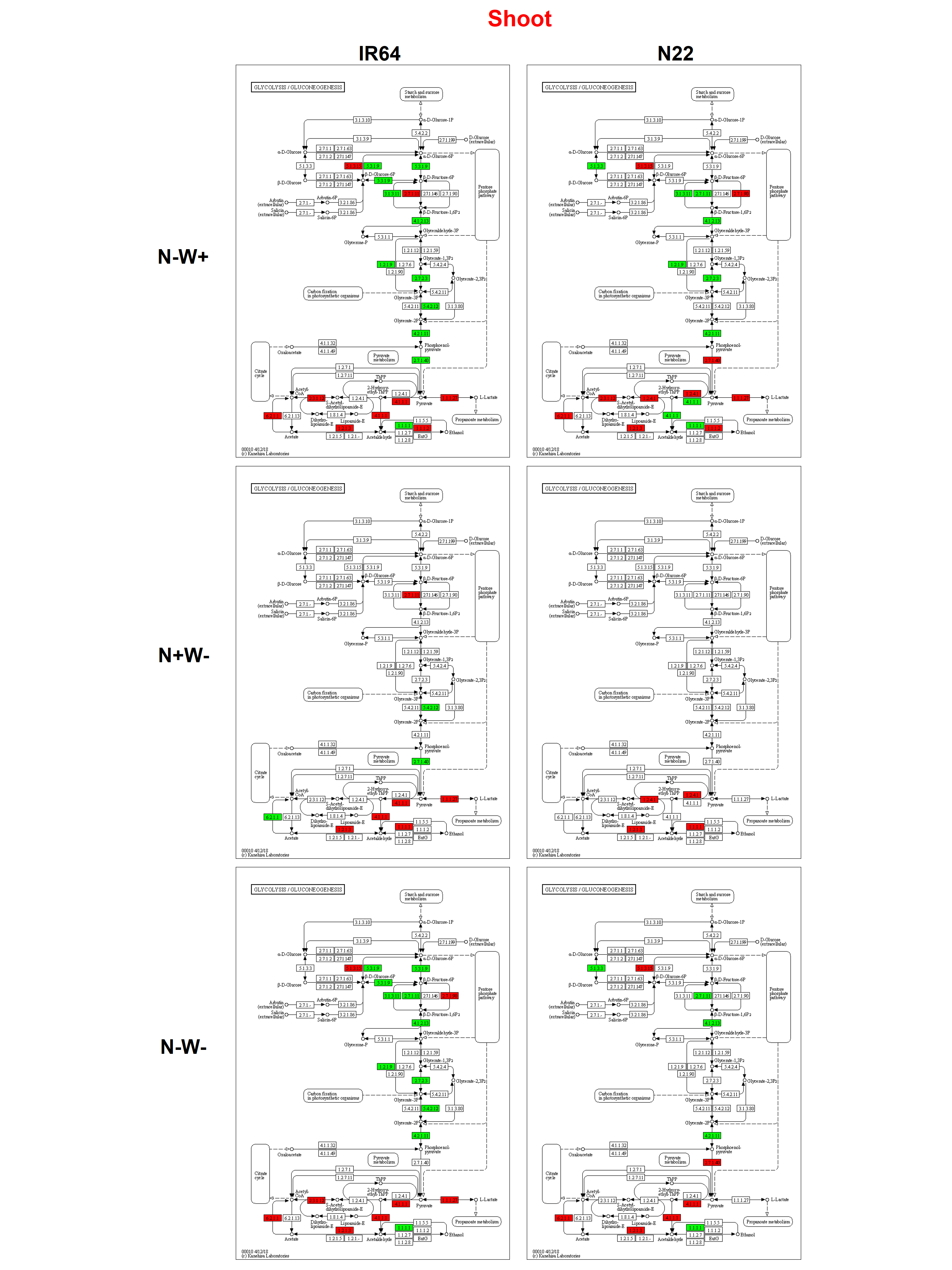

Supplement: Supplementary file 2 — Additional file 2: Supplementary Fig. 1: Pathway analysis of glycolysis in shoot tissues under low nitrogen (N-), low water (W-) and dual stress (N-W-) in IR64 and N22rice genotypes. [file 12284_2021_487_MOESM2_ESM.tif]

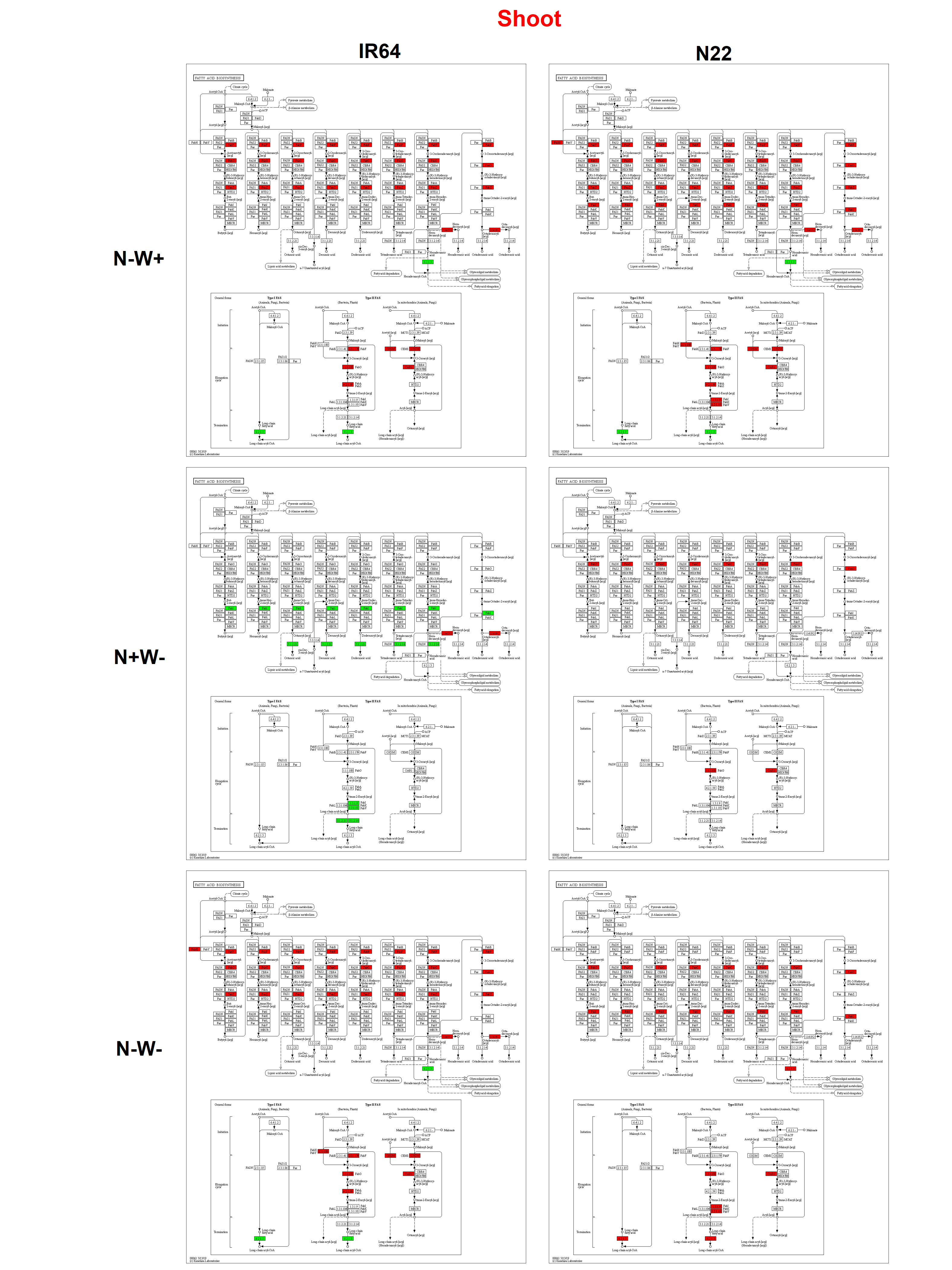

Supplement: Supplementary file 3 — Additional file 3: Supplementary Fig. 2: Pathway analysis of fatty acid metabolism in shoot tissues under low nitrogen (N-), low water (W-) and dual stress (N-W-) in IR64 and N22rice genotypes. [file 12284_2021_487_MOESM3_ESM.tif]

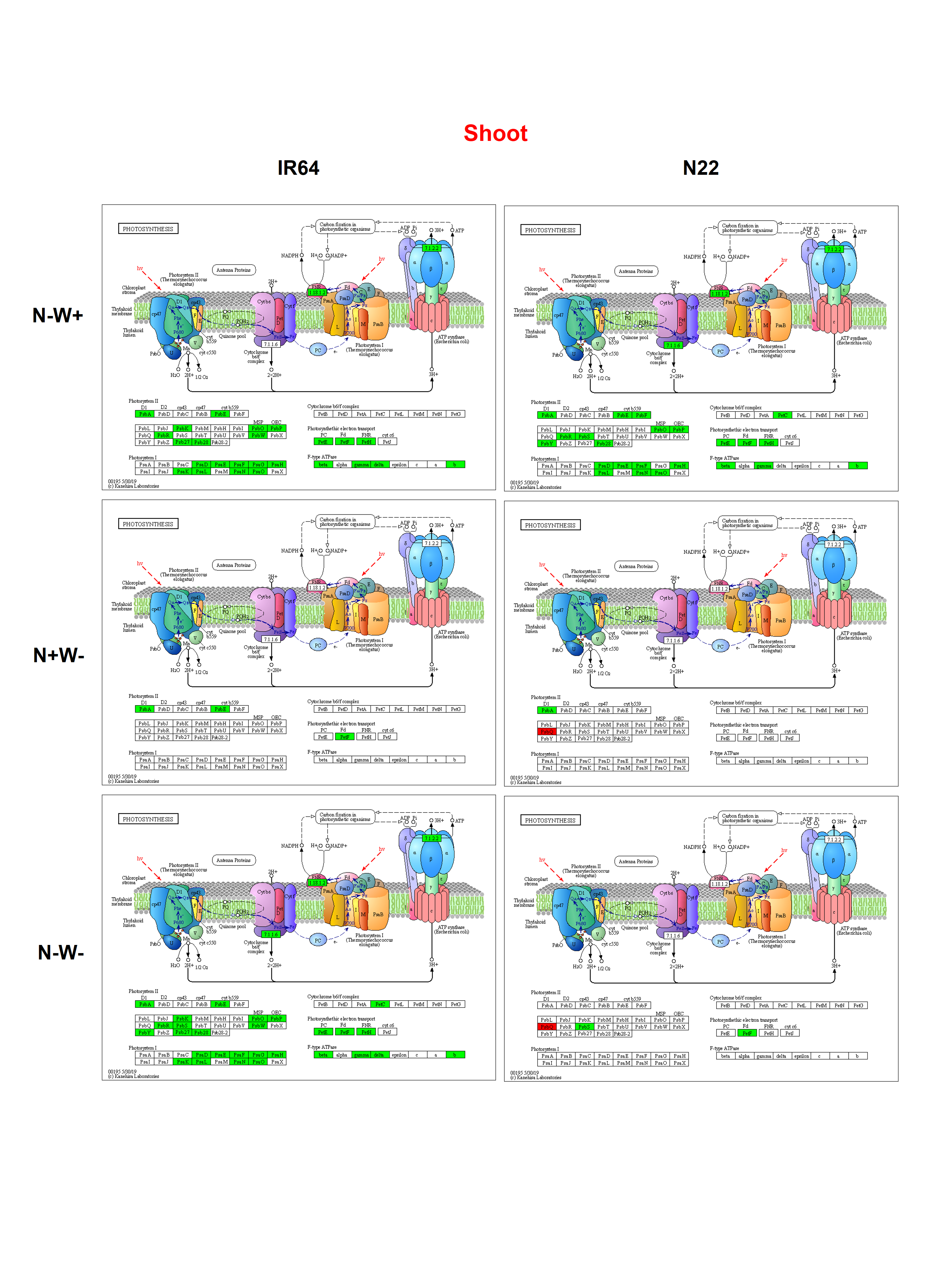

Supplement: Supplementary file 4 — Additional file 4: Supplementary Fig. 3: Pathway analysis of photosynthesis in shoot tissues under low nitrogen (N-), low water (W-) and dual stress (N-W-) in IR64 and N22rice genotypes. [file 12284_2021_487_MOESM4_ESM.tif]

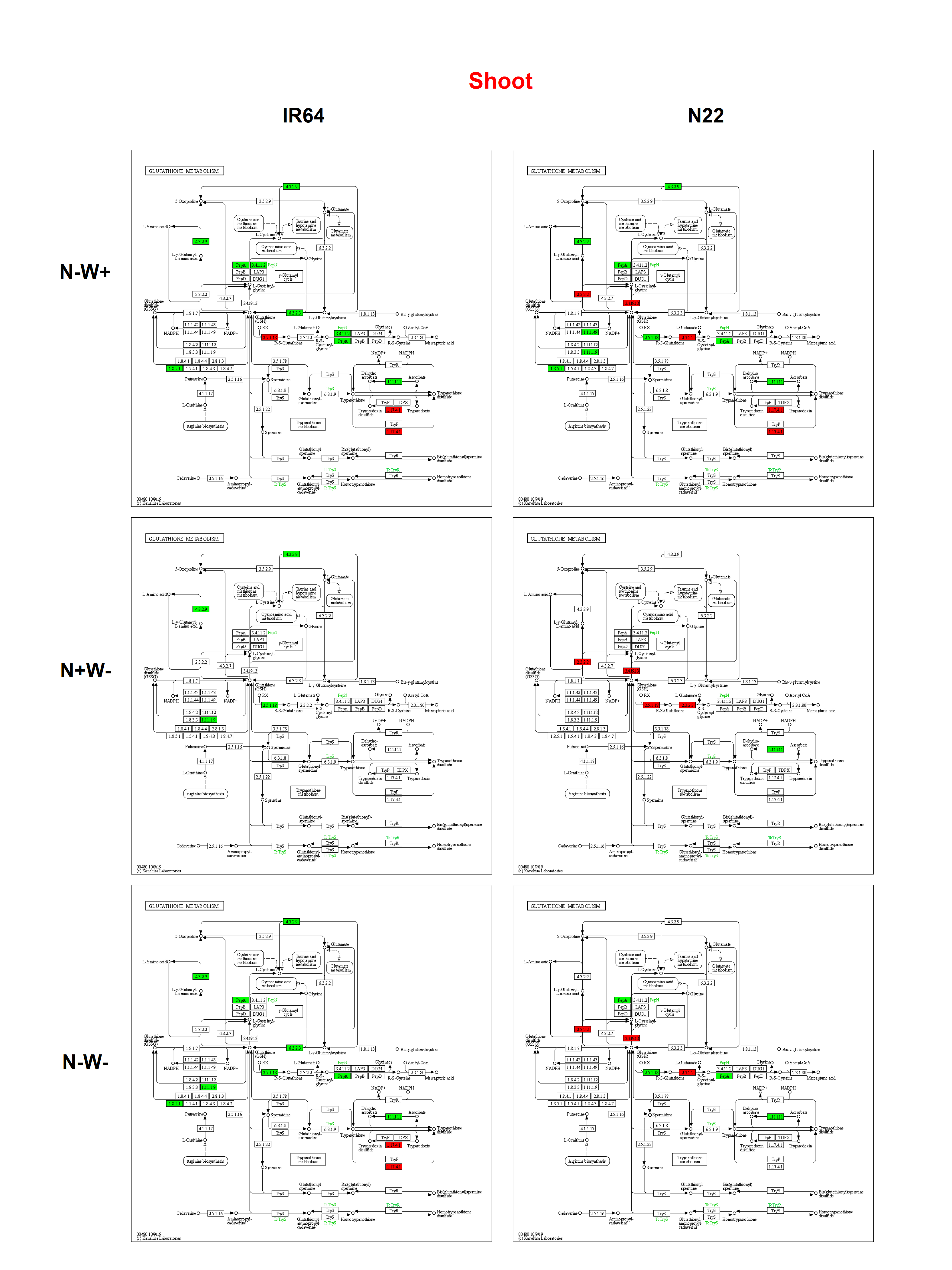

Supplement: Supplementary file 5 — Additional file 5: Supplementary Fig. 4: Pathway analysis of glutathione metabolism in shoot tissues under low nitrogen (N-), low water (W-) and dual stress (N-W-) in IR64 and N22rice genotypes. [file 12284_2021_487_MOESM5_ESM.tif]

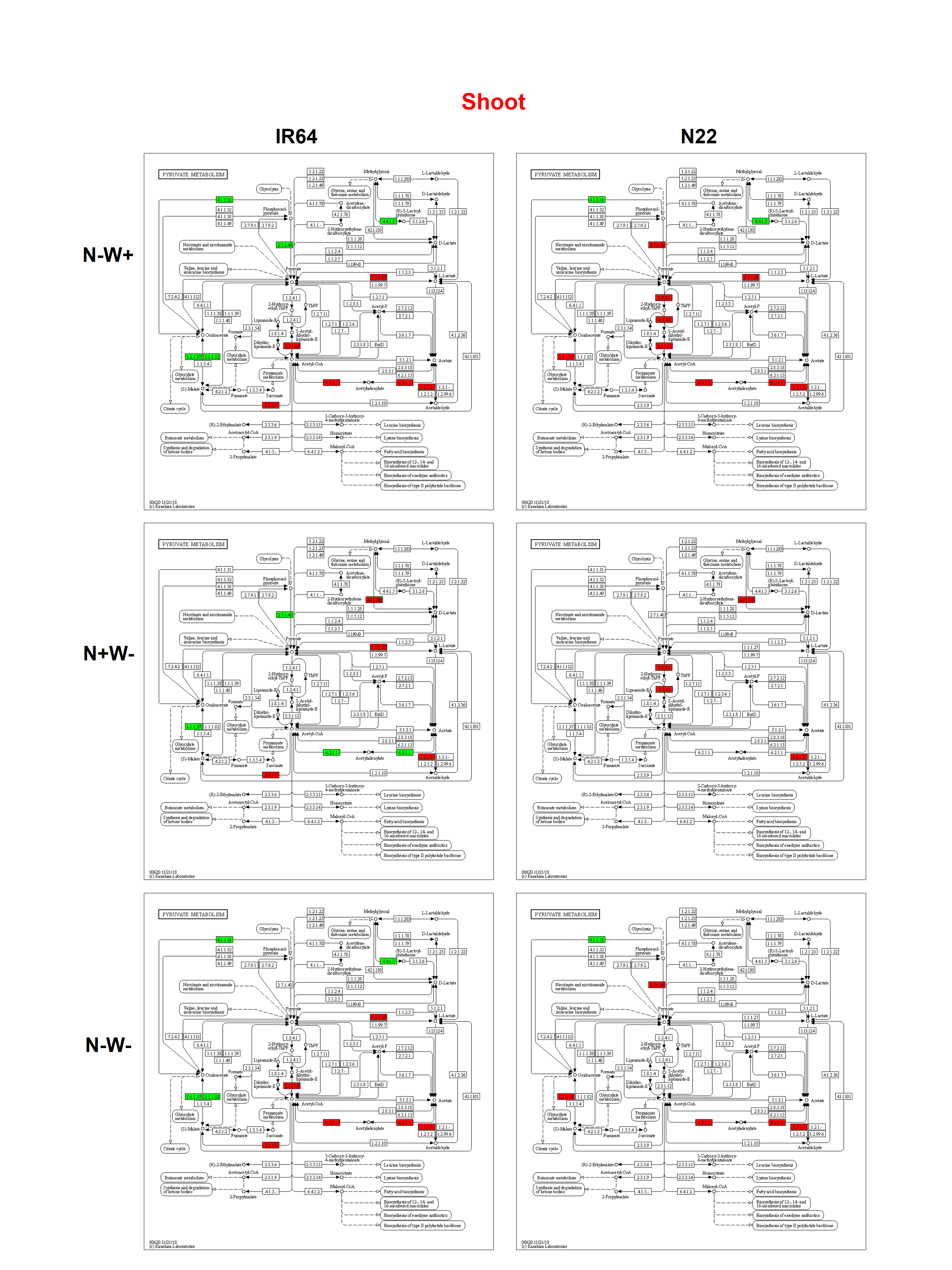

Supplement: Supplementary file 6 — Additional file 6: Supplementary Fig. 5: Pathway analysis of pyruvate metabolism in shoot tissues under low nitrogen (N-), low water (W-) and dual stress (N-W-) in IR64 and N22rice genotypes. [file 12284_2021_487_MOESM6_ESM.tif]

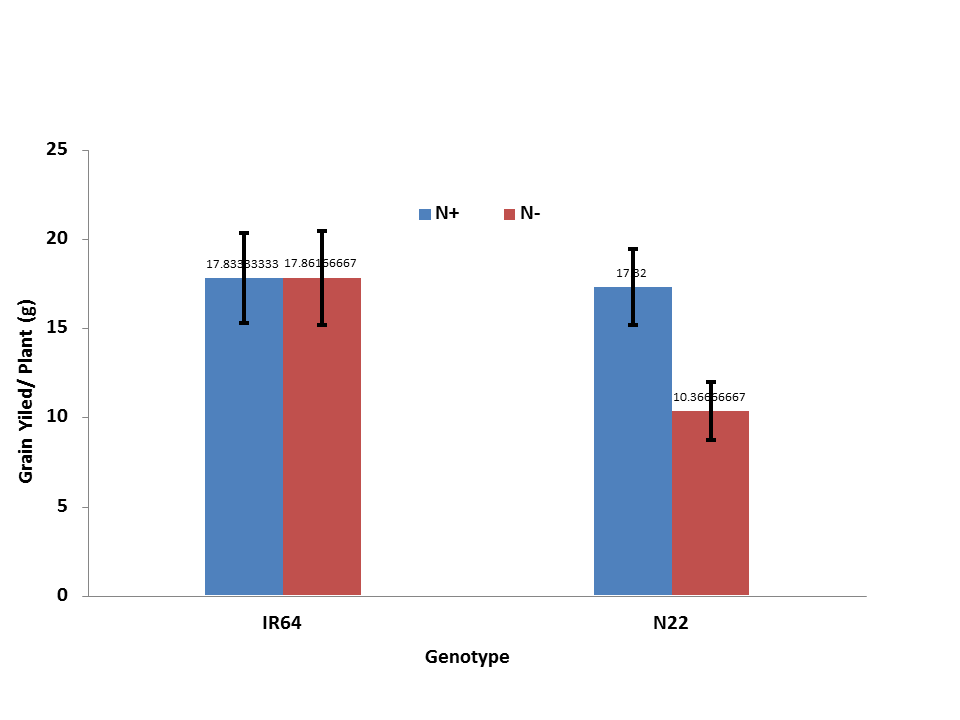

Supplement: Supplementary file 7 — Additional file 7: Supplementary Fig. 6: Yield of the two parental genotypes, IR 64 and Nagina 22 under N+ and N- plots. [file 12284_2021_487_MOESM7_ESM.tif]

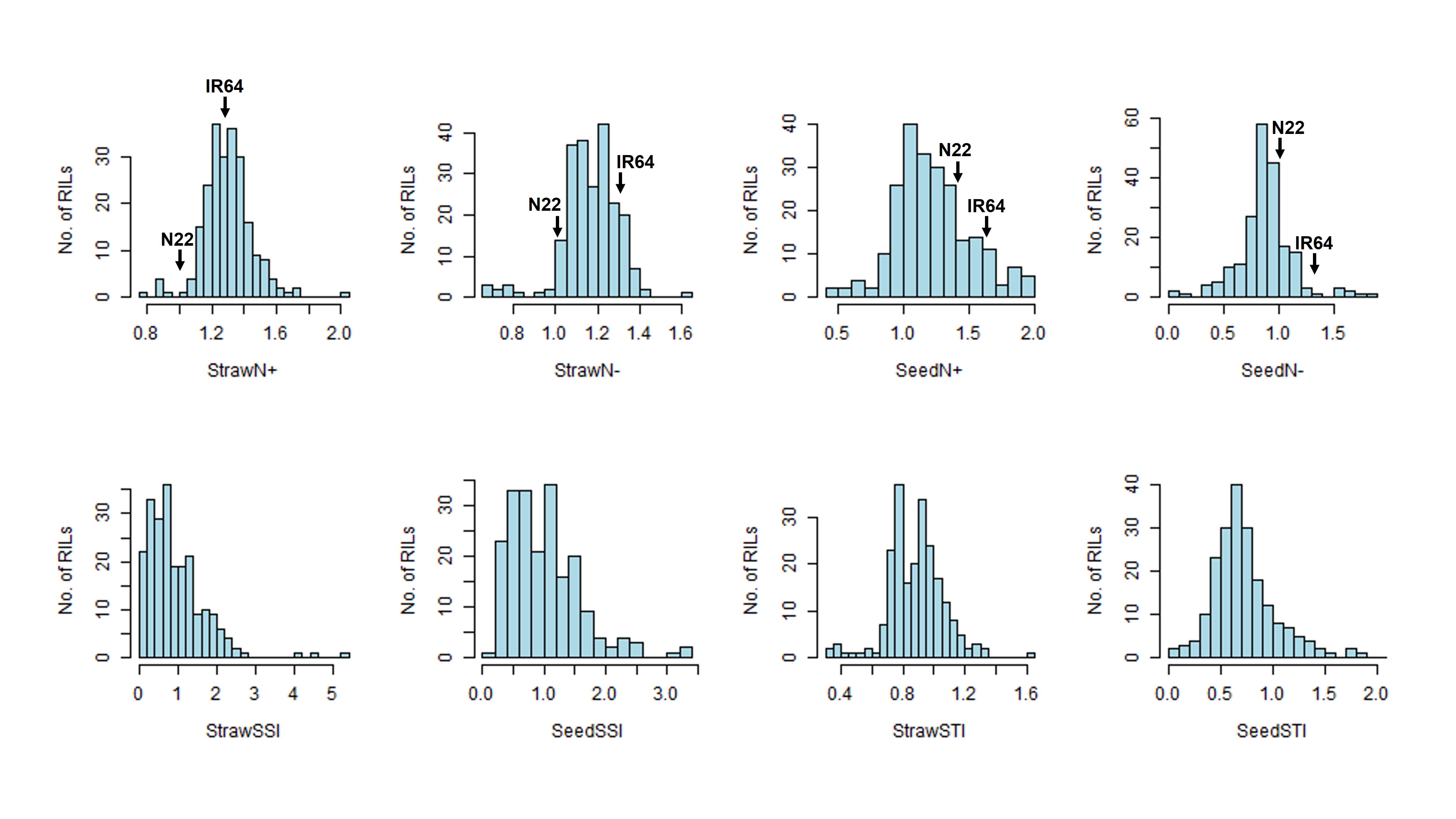

Supplement: Supplementary file 8 — Additional file 8: Supplementary Fig. 7: The frequency distribution of the eight traits based on straw and seed nitrogen content and their tolerant (STI) and susceptible (SSI) indices under no external N (NN) and optimal N (NP) supply. [file 12284_2021_487_MOESM8_ESM.tif]

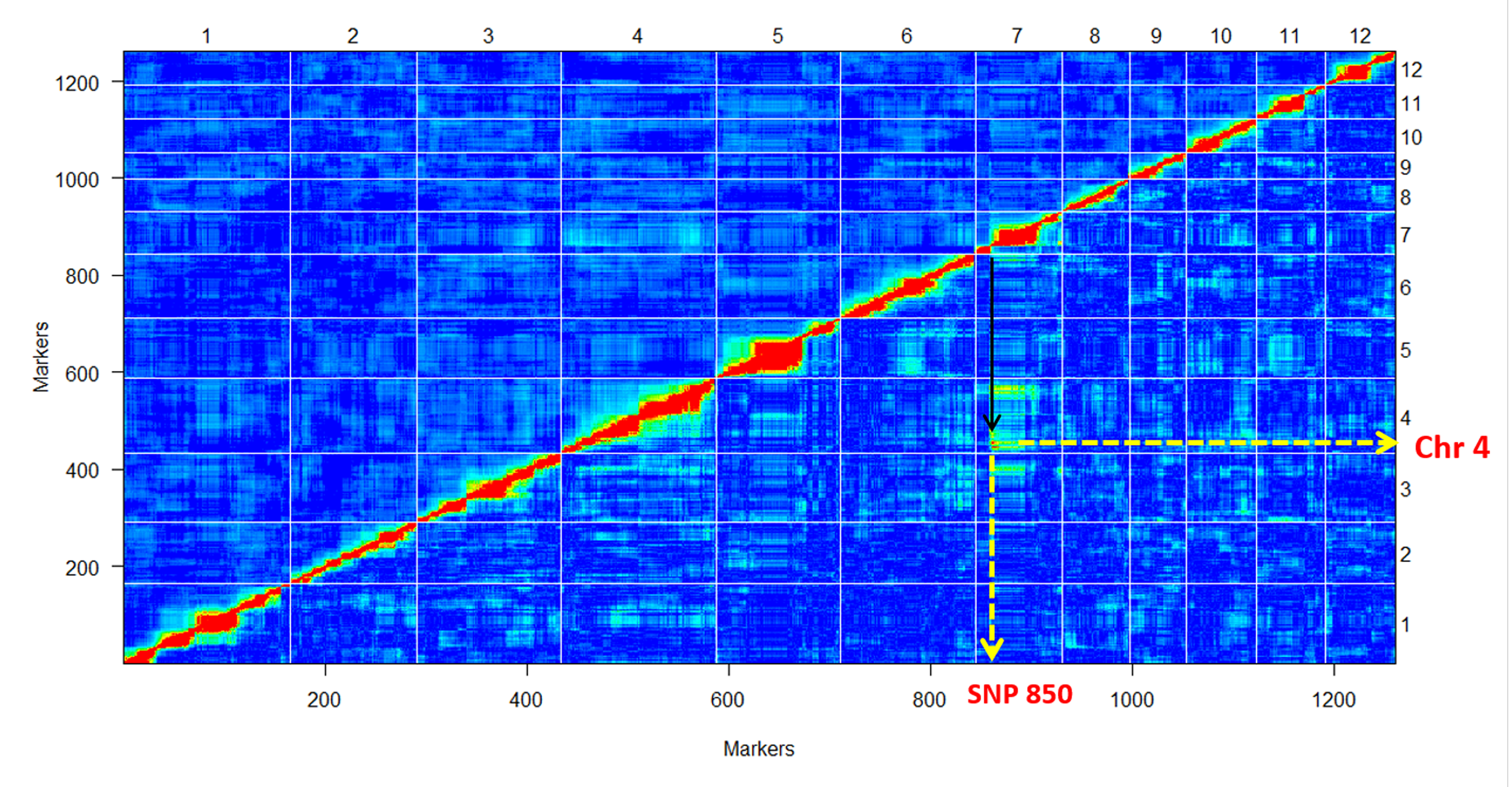

Supplement: Supplementary file 9 — Additional file 9: Supplementary Fig. 8: Quality assessment of polymorphic SNP markers between IR64 and N22 used for construction of genetic map and identification of QTLs in a recombinant inbred population. I-TASSER derived tertiary structure of the protein encoded by Os06g0289200 in N22 and IR64 genotypes. There were 28 SNPs and 11 non-synonymous substitutions between the CDS and primary structure of the protein in this gene between IR64 and N22. [file 12284_2021_487_MOESM9_ESM.tif]

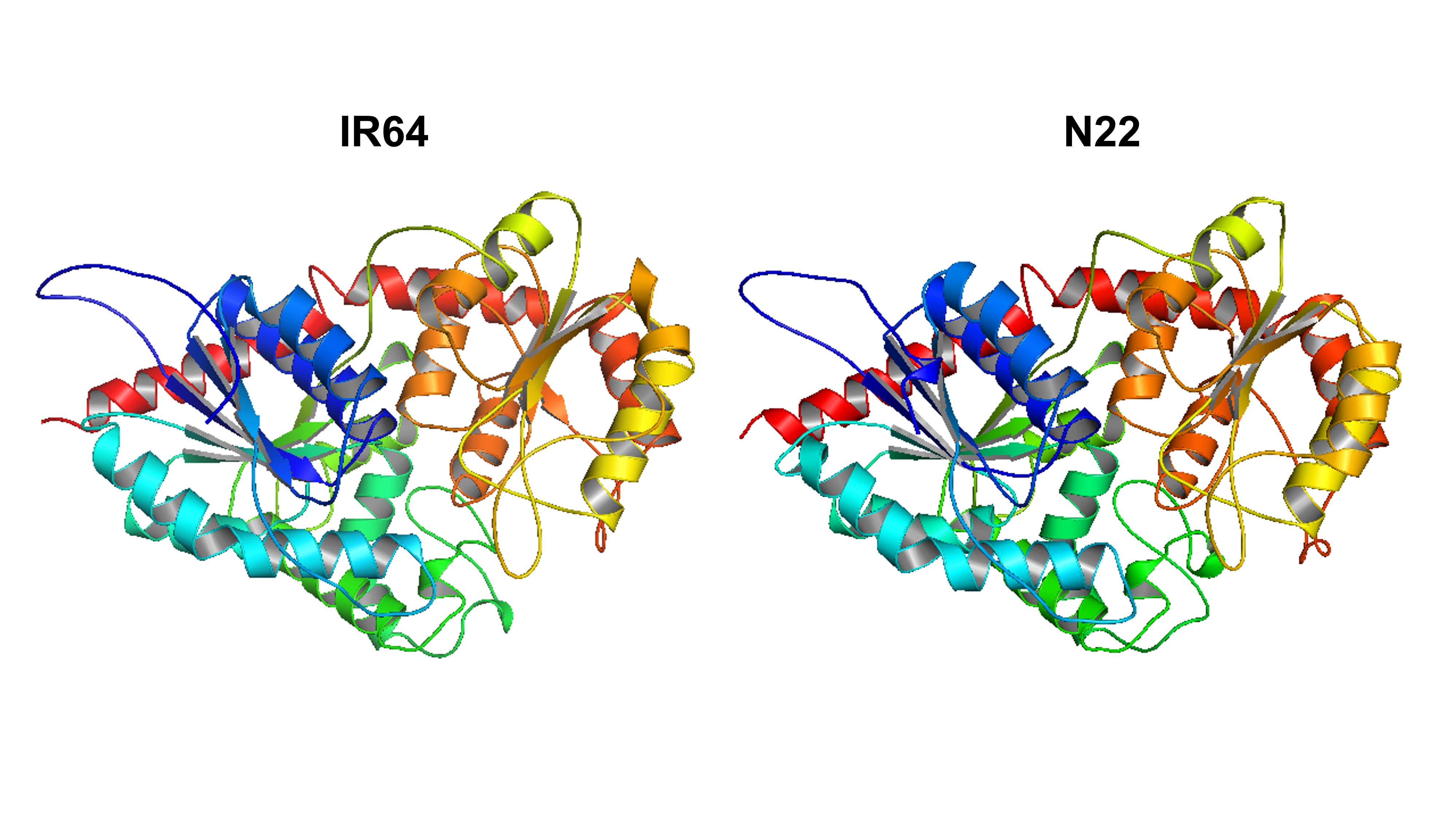

Supplement: Supplementary file 10 — Additional file 10: Supplementary 9: I-TASSER derived tertiary structure of the proteins encoded by Os06g0289200 in N22 and IR64 genotypes. There were 28 SNPs and 11 non-synonymous substitutions between the CDS and primary structure of the protein in this gene between IR64 and N22. [file 12284_2021_487_MOESM10_ESM.tif]
